# A novel salt-tolerant chitobiosidase discovered by genetic screening of a metagenomic library derived from chitin-amended agricultural soil

**DOI:** 10.1007/s00253-015-6639-5

**Published:** 2015-06-04

**Authors:** Mariana Silvia Cretoiu, Francesca Berini, Anna Maria Kielak, Flavia Marinelli, Jan Dirk van Elsas

**Affiliations:** Department of Microbial Ecology, CEES, University of Groningen, Groningen, The Netherlands; Department of Marine Microbiology, Royal Netherlands Institute for Sea Research, Yerseke, The Netherlands; Department of Biotechnology and Life Sciences, University of Insubria, Varese, Italy; “The Protein Factory” Research Center, Politecnico of Milano, ICRM CNR Milano and University of Insubria, Varese, Italy; Department of Microbial Ecology, The Netherlands Institute of Ecology (NIOO), Wageningen, The Netherlands

**Keywords:** Chitinolytic enzymes, Fosmid library, Functional metagenomics, Suppressive soil

## Abstract

Here, we report on the construction of a metagenomic library from a chitin-amended disease-suppressive agricultural soil and its screening for genes that encode novel chitinolytic enzymes. The library, constructed in fosmids in an *Escherichia coli* host, comprised 145,000 clones containing inserts of sizes of 21 to 40 kb, yielding a total of approximately 5.8 GB of cloned soil DNA. Using genetic screenings by repeated PCR cycles aimed to detect gene sequences of the bacterial chitinase A-class (hereby named *chi* A genes), we identified and characterized five fosmids carrying candidate genes for chitinolytic enzymes. The analysis thus allowed access to the genomic (fosmid-borne) context of these genes. Using the *chi*A-targeted PCR, which is based on degenerate primers, the five fosmids all produced amplicons, of which the sequences were related to predicted chitinolytic enzyme-encoding genes of four different host organisms, including *Stenotrophomonas maltophilia*. Sequencing and de novo annotation of the fosmid inserts confirmed that each one of these carried one or more open reading frames that were predicted to encode enzymes active on chitin, including one for a chitin deacetylase. Moreover, the genetic contexts in which the putative chitinolytic enzyme-encoding genes were located were unique per fosmid. Specifically, inserts from organisms related to *Burkholderia* sp., *Acidobacterium* sp., *Aeromonas veronii*, and the chloroflexi *Nitrolancetus hollandicus* and/or *Ktedonobacter racemifer* were obtained. Remarkably, the *S. maltophilia chi*A-like gene was found to occur in two different genetic contexts (related to *N. hollandicus*/*K. racemifer*), indicating the historical occurrence of genetic reshufflings in this part of the soil microbiota. One fosmid containing the insert composed of DNA from the *N. hollandicus*-like organism (denoted 53D1) was selected for further work. Using subcloning procedures, its putative gene for a chitinolytic enzyme was successfully brought to expression in an *E. coli* host. On the basis of purified protein preparations, the produced protein was characterized as a chitobiosidase of 43.6 kDa, with a pI of 4.83. Given its activity spectrum, it can be typified as a halotolerant chitobiosidase.

**Electronic supplementary material:**

The online version of this article (doi:10.1007/s00253-015-6639-5) contains supplementary material, which is available to authorized users.

## Introduction

Chitin and its derivatives are naturally occurring biopolymers, which are important for application in biomedicine, agriculture and the pharmaceutical industry. Particular features of chitins are their general biodegradability as well as lack of toxicity. Microbial enzymes active on chitin and chitin oligomers are of great interest for use in large-scale modification or degradation of chitin moieties. Two main areas of application have been described, i.e. (1) the development of biocontrol agents that allow to antagonize chitin-containing phytopathogenic fungi or nematodes for application in agriculture, and (2) the use of chitinolytic enzymes as industrial biocatalysts for the production of chitin derivatives.

Based on their mode of action, chitinolytic enzymes are classified into two categories: (1) chitinases (EC 3.2.1.14) that cleave the chitin chain at an internal site in a random manner, and (2) β-*N*-acetyl hexosaminidases (EC 3.2.1.52) that catalyze the successive removal of *N*-acetyl glucosamine residues from the non-reducing end of the chain (Adrangi and Faramarzi 2013). Chitinolytic enzymes belong to different families of glycosyl hydrolases (GH), whose classification is based on the amino acid similarity of their catalytic domains. A continuously updated list of these families is available through the ‘CAZy’ database (Cantarel et al. 2009). Most microbial chitinases belong to the family-18 GHs, whose catalytic domain is characterized by the presence of three highly conserved regions, [D/N]G[L/I/V/M/F][D/N][L/IV/M/F][D/N]xE, Y[D/N] and SxGG, the first two being involved in catalysis and the third one in substrate binding. The first signature pattern forms the β4 strand of the (β/α)_8_ triose-phosphate isomerase (TIM) barrel fold typically adopted by family-18 GHs (Watanabe et al. 1993). It contains the residues essential for the substrate-assisted double displacement hydrolysis mechanism, which includes the highly conserved glutamic acid residue proposed to be the catalytic proton donor (Watanabe et al. 1993). Family-18 GHs may have multi-domain structures with auxiliary regions, including carbohydrate-binding modules (CBMs) and fibronectin type III-like domains (FnIII), often found next to the catalytic domain (Eijsink et al. 2010). The presence of CBMs increases substrate-binding affinity and hydrolytic efficiency on crystalline chitin, while FnIII domains may be involved in altering the overall enzyme structure to facilitate the degradation of insoluble substrates (Vaaje-Kolstad et al. 2013; Toratani et al. 2006).

Analyses of sequence homologies have incited a division of the family-18 chitinases into the subfamilies A, B, and C (Suzuki et al. 1999; Li and Greene 2010; Eijsink et al. 2010). However, the nomenclature of bacterial chitinases does not follow this classification. For instance, the well-studied ChiA and ChiB from *Serratia marcescens* (Vaaje-Kolstad et al. 2013; Payne et al. 2012) both belong to subfamily A, while ChiC was classified in subfamily B (Suzuki et al. 1999). Subfamily B and C chitinases have been so far identified only in a few bacteria, while subfamily A ones are widespread in terrestrial and aquatic habitats (Cretoiu et al. 2012; Metcalfe et al. 2002; Li and Greene 2010). The latter encompass proteins with molecular weights of 20–115 kDa, optimal temperatures of 18–90 °C, pH of 2.0–10.5 and pI values of 3.5–8.0. They are further characterized by the presence of a small α + β domain (the chitin insertion domain—CID) present between the β7 and α7 of the TIM barrel catalytic domain (Li and Greene 2010; Eijsink et al. 2010). This CID forms a “wall” alongside the TIM barrel which increases the depth of the substrate-binding cleft, facilitating binding to long-chain substrates, and favoring the processive degradation of chitin (Li and Greene 2010; Zees et al. 2009; Vaaje-Kolstad et al. 2013).

Clearly, chitinolytic enzymes are prevalent in the microbiota of basically all ecosystems on Earth, with the highest quantities of chitin being turned over by microorganisms (bacteria and fungi) in marine and terrestrial settings (Beier and Bertilsson 2013; Delpin and Goodman 2009; Poulsen et al. 2008). Recently, the existence of an astonishing diversity of genes for predicted chitinolytic enzymes in natural microbiomes was unveiled (Beier and Bertilsson 2013; Kielak et al. 2013), and we are only at the beginning of understanding the significance of this diversity for ecological processes related to natural chitinolysis. It has been reported that functional diversity within the chitinolytic process is key to the functioning of the N cycle (Beier and Bertilsson 2013; Gooday 1990; Metcalfe et al. 2002; Manucharova et al. 2007). Furthermore, chitinolytic enzymes have been suggested to be involved in bacterial-fungal competition for plant root exudates in the rhizosphere (Bonfante and Anca 2009). Moreover, in agricultural soils, the addition of chitin helps to enhance the suppressiveness against soil-borne pathogens by stimulating active chitinolytic microbial communities (Korthals et al. 2010; Kielak et al. 2013; Cretoiu et al. 2013). Regarding the expression of chitinolytic enzymes in natural systems, a plethora of complex interactions may occur, with such enzymes often being produced as a result of sensing of the chitin substrate and/or intertwined with an environment-exploratory process, such as found for *Serratia plymuthica* (Khmel et al. 2005) as well as for the gliding bacterium *Flavobacterium johnsoniae* (Kharade and McBride 2014).

Recent developments in metagenomics-based analyses of the soil microbiota have enabled us to unlock the huge functional diversity, yielding access to novel genes and useful biomolecules (Ferrer et al. 2005; Simon and Daniel 2009; Nacke et al. 2011). Furthermore, the use of ecological enhancement (substrate enrichment) may increase the efficiency of mining for enzymes with improved features (Ekkers et al. 2012; Horn et al. 2012). Hence, a metagenomics approach with biased habitats to finding novel chitinolytic enzymes is warranted.

In the present study, we used a chitin-amended agricultural soil as the source of novel genes encoding predicted chitinolytic enzymes. We report the construction of a large-insert metagenomic library in fosmids in an *Escherichia coli* host. The library was subjected to PCR-based screenings using the degenerate primers previously described by Williamson et al. (2000) and Metcalfe et al. (2002) and more recently successfully used by Cretoiu et al. (2012) for mining unexplored habitats for novel chitinases. These primers have been previously shown to be selective for genes encoding putative bacterial chitinases belonging to subfamily A of family GH-18 (hereby named *chi*A-like gene sequences). Inserts of the thus-selected fosmids were then sequenced using high-throughput sequencing technology, after which the sequences were annotated. The gene for one selected predicted chitinolytic enzyme, provenient from a source organism related to the chloroflexi *Nitrolancetus hollandicus* and *Ktedonobacter racemifer*, was brought to expression in *E.coli*, after which the encoded protein was purified and its characteristics were determined.

## Materials and methods

### Soil samples

Soil samples were collected from a chitin-treated agricultural field located at the experimental farm “Vredepeel,” the Netherlands. The field has been used since 1990 by Applied Plant Research (PPO) to monitor the effects of diverse agricultural practices. The soil, a loamy sand (pH-KCl 5.7 ± 0.2, 3.2 % organic matter), was supplemented with 1.8 % of shrimp waste chitin (20 tons/ha) calculated over the topsoil (20 cm). The soil was sampled 9 months after chitin amendment. Details can be found in Cretoiu et al. (2013).

### Microbial community DNA extraction

Extraction of soil DNA for the construction of a metagenomic library was performed using a modification of a previous protocol (Van Elsas et al. 2008). Briefly, 10 g of soil were suspended in 10 ml buffer (100 mM Tris-HCl, 100 mM NaEDTA, 100 mM NaPO_4_, 1.5 % NaCl, 1 % CTBA, pH 8.0), shortly Vortex-mixed and sonicated (water bath) for 15 min. After sonication, 100 μl of proteinase K (10 mg/ml) were added, which was followed by incubation at 37 °C (2 h) with shaking at 200 rpm. DNA was gently extracted with phenol/chloroform/iso-amylalcohol (25:24:1) at 60 °C for 30 min. The DNA was then precipitated with 2-propanol, dissolved and embedded in plugs of (1 %) low-melting-point agarose. Then, 30–40 kb DNA fragments were separated by pulsed-field gel electrophoresis (PFGE) at 14 °C on 1 % agarose gels supplemented (upper part) with 2 % polyvinyl pyrrolidone (PVP), in 0.5 strength Tris-borate-EDTA (TBE). A PFGE DRIII system (BioRad, CA, USA) was used with gradient of 6 V/cm, included angle 120^o^, initial switch time 0.5 s, final switch time 8.5 s, linear ramping factor 20 h. Agarose fragments (2 cm) containing the 30–40 kb DNA fragments were then excised from gel, after which a β-agarase (New England Biolabs, MA, USA) treatment allowed recovery of the DNA.

### Fosmid library construction

Library construction was performed in the pCC1Fos vector (Epicentre, Madison, WI, USA). The 30–40 kb DNA was 5′-phosphorylated/blunt-ended, after which it was ligated into pCC1Fos. *E. coli* EPI300-T1^R^ cells (Epicentre) were then transformed with the ligation mixes and plated on Luria-Bertani (LB) agar supplemented with 12.5 μg/ml chloramphenicol (LB + Cm). Plates were incubated overnight at 37 °C and colonies pooled, after which the pools were stored at −80 °C.

### Presumptive identification of genes for chitinolytic enzymes

We used a “degenerate” PCR system for the detection of putative genes encoding bacterial chitinases of subfamily A of the GH-18 family, as developed in Williamson et al. (2000). This system was shown to uncover a huge diversity of *chi*A-like genes (that are classifiable among a wide range of bacterial species) from soil, as shown in a recent study by Kielak et al. (2013). The amplicons, expected to be 0.45–0.6 kb in size, as described in detail by Cretoiu et al. (2012), were visualized by standard agarose gel electrophoresis. Whenever detected, amplicons were extracted from gel using Wizard SV gel and PCR CleanUp systems (Promega, Madison, WI, USA) and sequenced using the reverse primer (LGC, Berlin, Germany). Strain EPI300-T1^R^ and vector pCC1Fos DNAs, used as controls, never yielded any positive amplification.

### Screening for genes encoding predicted chitinolytic enzymes

Library clones were screened for the presence of c*hi*A-like genes using a “pool-subpool-single” PCR strategy (Israel 1993; Peterson et al. 2002). Pooled, sub-pooled, or single fosmid clones were cultured overnight in chloramphenicol-amended Luria-Bertani broth (LB + Cm) in 96-well plates. The contents of two plates (192 clones) were combined for extractions using QIAprep Spin Miniprep kits (Qiagen, Venlo, The Netherlands). The resulting DNA preparations were used for the PCR screenings. In cases of positive reactions, sub-pools consisting of the rows of each plate were tested, and the positive result was then further reduced to the single-clone level. At “single-row” and “single-clone” levels, fosmid copy numbers per cell were raised by adding 0.4 μl of auto-induction solution (500X) per 200 μl LB + Cm (Epicentre). Amplicons were analyzed by agarose gel electrophoresis, after which the presence of *chi*A*-*like sequences was assessed by sequencing. Resulting sequences were analyzed by tBLASTx. Then, they were aligned with 22 selected sequences encoding well-characterized family-18 GHs (retrieved from GenBank and CAZy), by using Clustal-W (BioLinux7; Field et al. 2006). Phylogenetic reconstruction was based on neighbor-joining, using bootstrapping (100 repetitions) and a nucleic acid substitution model (MEGA 5.2).

### DNA extraction from selected fosmid clones

Fosmid clones selected based on the presence of *chi*A-like sequences were cultured in 2 ml LB + Cm and copy numbers raised by autoinduction (overnight, 37 °C). DNA was then extracted using the Gene Jet Plasmid Midi Preparation Kit (ThermoFisher Scientific, St. Leon-Rot, Germany), followed by PFGE analyses as above. DNA concentration was measured by spectrophotometry (Nanodrop; ThermoFisher Scientific).

### Sequencing of fosmid insert DNA

The inserts of selected fosmid clones were fully sequenced at BaseClear (Leiden, The Netherlands). Prior to sequencing, DNA concentration and quality (the ‘integrity number’) were assessed by microfluidics-based electrophoresis (Bioanalyzer, Agilent Technologies, Waldbronn, Germany). Paired-end libraries were prepared for each fosmid (Paired-End DNA Sample Preparation Kit, with specific adaptors; Illumina, Eindhoven, The Netherlands), after which sequencing ensued (HiScanSQ Illumina system).

### Sequence assembly and analysis

Processing of raw sequence data (BaseClear, Leiden, The Netherlands), implying the generation of FASTQ sequence reads, quality control and de novo assembly, yielded—for each fosmid—robust contiguous sequences (contigs). Briefly, FASTQ reads (Illumina Casava pipeline; version 1.8.2), were quality-assessed using the Illumina Chastity filtering system. Then, reads containing adapters and/or PhiX control signal were removed (*in house* filtering). The remaining reads were subjected to the in-house FASTQC quality control tool (version 0.10.0). Then, low-quality bases were removed using “Trim sequences” (CLC Genomics Workbench v. 5.5.1; CLC Bio, Aarhus, Denmark), and vector sequences further eliminated. Finally, contigs were formed using all quality-controlled sequences by de novo assembly (CLC Genomics Workbench). Contigs were accepted if average coverage values were >500-fold. Coverage was calculated by mapping reads against contigs.

### Annotation of contig sequences

All final contigs, representing the fosmid inserts, were checked for the presence of *chi*A-like gene sequences. Thus original amplicon sequences were aligned against the full-length contigs (BioEdit v. 7.2.0 sequence alignment editor; Hall 1999), and manually checked for errors and gaps. Open reading frames (ORFs) were then assigned by using GLIMMER (v.3.02; Delcher et al. 1999; http://cbcb.umd.edu/software/glimmer/) on a BioLinux v.7 platform (Field et al. 2006), followed by MetaGene (Noguchi et al. 2006) and RAST (National Microbial Pathogen Data Resource, NMPDR) confirmation (Aziz et al. 2008). The RAST annotations yielded predicted protein-encoding genes (coding DNA sequences (CDSs)). These were manually curated and verified by similarity searches against the non-redundant protein database (http://www.ncbi.nlm.nih.gov) using BLAST-P (http://blast.ncbi.nlm.nih.gov/Blastp) with optimized parameters, as in Table S1A (http://www.ncbi.nlm.nih.gov/guide/training-tutorials/ BLAST tutorials and guides/). The nearest protein homologs were determined, as in Rost (1999) and Raghava and Barton (2006), taking into account query coverage (in percent), maximum identity (in percent), alignment scores (maximum and total score) and *e* value (Table S1B). RAST annotation also included a scan for tRNA genes and classification according to the “Cluster of Orthologous Groups” of proteins (COGs). ORFs <120 bp were discarded when query coverage and maximum identity criteria were not in the required range. Intergenic regions were searched against the non-redundant database (http://www.ncbi.nlm.nih.gov) using all BLAST options to ensure that no ORF was missed. Start and stop codons were identified for all annotated ORFs.

### Prediction and phylogenetic analysis of genes encoding putative chitinolytic enzymes

Prediction of genes encoding putative chitinolytic enzymes/family-18 GH proteins was performed using the InterProScan (EMBL) integrative tool (search in all available functionally annotated protein databases, using sequences of single proteins, protein superfamilies and hidden Markov models) (Quevillon et al. 2005). Protein domain organization was predicted using InterPro Scan5 (http://www.ebi.ac.uk/Tools/pfa/iprscan5/) and ExPASy Prosite (http://prosite.expasy.org/). Putative signal peptides were searched with PRED-TAT (Computational Genetics Group, http://www.compgen.org/tools/PRED-TAT/submit), SignalP 4.1 (CBS Technical University of Denmark, http://www.cbs.dtu.dk/services/SignalP/) and TatP 1.0 (CBS Technical University of Denmark, http://www.cbs.dtu.dk/services/TatP/). Furthermore, secondary and tertiary structures of proteins were predicted on the ITASSER server (Roy et al. 2010; http://zhanglab.ccmb.med.umich.edu/I-TASSER/) using default parameters. Bacterial promoters were predicted using BProm (SoftBerry, http://linux1.softberry.com/berry). Ribosome-binding sites (RBS) were identified using the RBS calculator (https://salis.psu.edu/software/; Salis et al. 2009) and manually checked as in Shultzaberger et al. (2001) and Stewart et al. (1998). The taxonomic affiliations of genes identified as encoding a chitinolytic/family-18 GH protein were confirmed by comparison with CAZy entries using Mothra.ornl (CAZYmes analysis toolkit; Park et al. 2010). All identified gene sequences were then subjected to Clustal-W (BioLinux v. 7; Field et al. 2006), along with sequences of genes for 65 defined chitinolytic enzymes (CAZy database). Phylogenetic reconstruction was performed by the Maximum Likelihood (ML) algorithm using the model of Jones–Taylor–Thornton (JTT) with uniform rates, partial deletion and site coverage cut-off of 95 % and bootstrapping with 100 replications. A characterized cellulase gene of *E.coli* P12b was used as the outgroup sequence.

### Prediction of the origin of fosmid inserts and of potential horizontal gene transfer

The origins of the fosmid inserts were predicted based on the phylogenetic affiliation of >75 % of the identified ORFs. We interpreted these with the cautionary note in mind that de novo annotation is questionable for soil-derived sequences (Raes et al. 2007). Synteny of the recovered fosmid inserts with regions of existing genomes and intergenic region similarities was determined using “Mauve” (Darling et al. 2010). Searches for G + C-rich islands were performed using CpGFinder (SoftBerry; http://linux1.softberry.com/berry). Nucleotide frequency analyses were performed for screening of potentially horizontally transferred regions (Scater plot viewer, http://www.jcvi.org/cms/research/). ScaterPlot was available from J. Craig Venter Institute Platform Informatics, and allowed searching for tetranuclotide patterns, considering every possible option, i.e., AAAA, AAAT, AAAC, AAAG, AATT, etcetera, giving 4^4 = 256 possibilities.

### Sub-cloning of the gene for a presumptive chitinolytic enzyme carried by fosmid 53D1

*E.coli* DH5α (Invitrogen Life Technology, Carlsbad, USA) was used for all cloning procedures. A DNA region encoding the fosmid 53D1 *chi*A-like sequence was obtained by PCR. The amplicons were cloned into expression vector pET24b(+) (Novagen Inc., Madison, USA), with addition of a polyhistidine tag (His_6_-Tag) at the C-terminus. Primers 53D1_pET24b_FW (5′-ACCACATATGATGAGTCACGGTTCGGTCTCTCC-3′) and 53D1_pET24b_RV (5′-AATACTCGAGCGGTCTCAGCCGGGATGAGA-3′), containing restriction sites for *Nde*I and *Xho*I respectively, were used. Also, cloning into the pColdI vector (TaKaRa Bio Inc., Otsu, Japan) was performed, with the His_6_-Tag added at the N-terminus of the protein. Here, primers 53D1_pColdI_FW (5′-AATTGAGCTCAGTCACGGTTCGGTCTCTCC-3′) and 53D1_pColdI_RV (5′-CCAAAAGCTTTTACGGTCTCAGCCGGGATG-3′) were used. These contained restriction sites for *Sac*I and *Hind*III, respectively. To study gene expression under the control of the putative native promoter, additional primers were designed to amplify a 150-bp region upstream of the predicted start codon. These were: 53D1prom_pET24b_FW (5′-AATACATATGCGGTCGGATGACTGTGGCGCC-3′) and 53D1prom_pET24b_RV (5′- AATACTCGAGCGGTCTCAGCCGGGATGAGA-3′), carrying, respectively, *Nde*I and *Xho*I restriction sites*.* All gene products were verified by DNA sequencing (BMR Genomics, Padova, Italy). *E. coli* BL21 Star^TM^(DE3) (Invitrogen, Carlsbad, USA) transformed with pColdI::*53D1* or pET24b(+)::*53D1*, and *E. coli* DH5α carrying pET24b(+)::*53D1prom* plasmids were maintained on LB agar supplemented with 50 μg/ml kanamycin (selective for the latter strain) or 100 μg/ml apramycin (selective for the former).

### Expression and purification of the fosmid 53D1-encoded putative chitinolytic enzyme

Experiments with the three strains described above and with controls carrying empty vectors were conducted in LB medium and in ‘Terrific Broth’ (TB, Sigma-Aldrich, St Louis, USA). For protein purification, early-exponential *E. coli* BL21 Star^TM^ (DE3)/pET24b(+)::*53D1* cells in LB (OD_600nm_ ~ 0.6) were treated with 0.5 mM isopropyl β-d-thiogalactopyranoside (IPTG) and harvested after overnight incubation at 25 °C (shaking, 200 rpm). After washing with sodium chloride-Tris-EDTA (STE buffer: 10 mM Tris-HCl pH 8.0, 1 mM EDTA, 100 mM NaCl), 3 g of cell paste (obtained from 550 ml culture) were sonicated on ice (5 cycles, 30 sec each). His_6_-53D1 protein was purified from the soluble cytoplasmic fraction (added to 500 mM NaCl) by loading onto 5 ml Ni^2+^-Hitrap chelating affinity columns (GE Healthcare Sciences, Little Chalfont, UK), equilibrated with 50 mM Tris-HCl (pH 8.0), 500 mM NaCl and 20 mM imidazole. The protein was eluted with increasing concentrations of 50 mM Tris-HCl (pH 8.0), 500 mM NaCl and 250 mM imidazole, and loaded onto a size-exclusion PD10 Sephadex G25 column (GE Healthcare Sciences, Little Chalfont, UK) equilibrated with 50 mM Tris-HCl (pH 8.0). The yield was 0.638 mg/l culture (0.117 mg/g cells).

### SDS-PAGE electrophoresis, Western blot, and zymogram

Protein purity was checked by sodium dodecyl sulfate-polyacrylamide (12 % *w/v*) gel electrophoresis (SDS-PAGE) (Schagger and van Jagow 1987). For Western blot analysis, the protein was identified by anti His-Tag Antibody HRP conjugate (Novagen Inc., Madison, USA) as detected by chemiluminescence (ECL Western Blotting Detection System, GE Healthcare Sciences, Little Chalfont, UK). Molecular weight markers were from the latter. A zymogram was used to detect chitinolytic activity on polyacrylamide gel (10 % *w/v*) containing 0.7 mg/ml carboxymethyl-chitin-remazol brilliant violet (CM-chitin-RBV) (Loewe Biochemica, Sauerlach, Germany) as in Hjort et al. (2014).

### Determination of chitinolytic activity

Chitinolytic activity was fluorimetrically assayed with the chitooligosaccharide analogues 4-methylumbelliferyl *N*-acetyl-β-d-glucosaminide (4-MU-GlcNAc), 4-methylumbelliferyl *N*,*N′*-diacetyl-β-d-chitobioside (4-MU-(GlcNAc)_2_) and 4-methylumbelliferyl *N,N′,N″*-triacetyl-β-d-chitotrioside (4-MU-(GlcNAc)_3_) as substrates, as in Hjort et al. (2014). One unit (U) of activity was defined as the amount of enzyme required for the release of 1 μmole 4-MU per min at pH 5.0 and 37 °C. Chitinolytic activity on colloidal chitin (from shrimp shells; Sigma-Aldrich, St Louis, USA), prepared as described (Hsu and Lockwood 1975), was determined by a colorimetric method (Anthon and Barrett 2002). Briefly, 250 μl of sample was added to 250 μl of 10 g/l colloidal chitin, and the mixture was incubated (37 °C, 1 h), boiled for 5 min and centrifuged (20,000×*g*, 25 °C, 15 min). Then, 200 μl of supernatant was mixed with 200 μl each of 0.5 M NaOH and of 3-methyl-2-benzothiazolinone hydrazone (MBTH). After 15 min at 80 °C, 400 μl of 0.5 % (*w/v*) FeNH_4_(SO_4_)_2_ .12H_2_O/0.5 % (*w/v*) sulfamic acid/0.25 M HCl was added, allowing the mix to cool to room temperature. After addition of 1 ml of H_2_O, absorbance at 620 nm was determined. Released reducing sugars were estimated by comparison to a standard curve prepared with an *N-*acetyl-d-glucosamine concentration range (0–600 μM). One U of chitinolytic enzyme activity was defined as the amount of enzyme that released 1 μmol/ml *N-*acetyl-d-glucosamine per h at 37 °C.

### Enzyme characterization

The optimum pH for 53D1 enzyme activity was determined by the fluorimetric assay (see above) with the following buffers (100 mM) at the respective pH: glycine-HCl (pH 3.0), sodium acetate (pH 4.0 and 5.0), sodium phosphate (pH 6.0 and 7.0), Tris-HCl (pH 8.0), and sodium pyrophosphate (NaPPi, pH 9.0). The optimum temperature for 53D1 enzyme activity was determined by incubating reaction mixtures at temperatures of 5–70 °C. The effects of metal ions (20 mM), enzyme inhibitors (5 % *v/v*), chelating agents (20 mM), detergents (1 % *w/v*), organic solvents (10 % *v/v*), sugars (10 mM *N-*acetyl-d-glucosamine and 10 mM chitobiose) and NaCl levels (up to 2 M) were investigated by adding each compound to the assay mixtures. All reagents were from Sigma-Aldrich, St Louis, USA.

## Results

### Construction of a metagenomic fosmid library from chitin-treated field soil

We used soil sampled from a long-term chitin-treated field soil, in which important changes in soil disease suppressiveness (e.g., resistance to *Verticillium dahliae*; Korthals et al. 2010), as well as in particular soil bacterial communities, had been detected (Cretoiu et al. 2013). Using 10 g of this chitin-amended soil, we obtained 0.25 μg of DNA per g soil, with an average fragment size of about 40 kb. The resulting DNA was found to be pure enough to serve for direct cloning into the Epicentre fosmid system. Thus, a library encompassing 145,000 insert-containing fosmid clones in *E. coli* was generated. The resulting cells were harvested into pools that each contained up to roughly 1,500 individuals. The estimated size of the collective inserts was 5.8 GB, which is comparable to the size of other large soil metagenomic libraries (Nacke et al. 2011).

### Screening for *chi*A-related genes

Family-18 GH chitinase genes were recently shown to have high sequence variability (Kielak et al 2013) that were all captured using degenerate primers initially designed on the basis of the conserved motifs present in the extended catalytic domains (Williamson et al. 2000, refer also to the “Introduction” section). Thus this broad *chi*A-based PCR screening method was used to detect potentially novel ‘*chi*A*-*like’ gene sequences. All 100 fosmid pools were thus screened in order to detect the broadest possible range of presumptively *chi*A-positive fosmids. In total, 18 fosmids yielded consistently positive PCR results (screened twice). The resulting amplicons, of 450–600 bp in size, were cloned and subjected to sequence analysis, after which the sequences were compared to those of selected genes that encode well-described chitinolytic proteins by BLAST-N comparisons. Thirteen of the 18 sequences were very remote from any retrieved *chi*A-like gene sequences, revealing <35 % nucleotide sequence homology. Thus, we subjected the remaining five pools (each containing predicted genes with homologies >35 % to known chitinases) to several cycles of splitting up in sub-pools and PCR monitoring, finally isolating single *chi*A-like gene containing fosmids. Sequencing of the amplicons of each of the five clones confirmed these as *chi*A-related genes, that resembled GH-18 genes from *Stenotrophomonas maltophilia*, *Niastella koreensis* GR20-10, *Streptomyces coelicolor* A3(2), and *Kitasatospora setae* KM-6054 (next to other organisms). The fosmid inserts were subjected to further characterization using functional analysis and whole insert sequencing, as detailed in the following.

### De novo annotation and general characteristics of genetic fragments recovered from fosmids of the metagenomic library

The five “*chi*A-positive” fosmid clones, denoted 14A, 22G3, 28C5, 53D1 and 101F8, did not reveal expression of chitinolytic activity using the fluorimetric assay on three chitooligosaccharide analogues (performed as in Hjort et al. 2014). To understand the genomic context of the *chi*A homolog per fosmid, the inserts of all five fosmids were subjected to full sequencing. The paired reads per fosmid amounted to 6.4–9.6 Mb of sequence information (Table S2A). The sizes of the assembled inserts were in the range 21.2–37.9 kb, with G + C contents revolving around 58.8 (±6.4) %. The latter metric was specific per fosmid, as detailed below. Moreover, for each fosmid it was consistent across the full length of insert (Table S2B). Overall, tetranucleotide counts (TNC) varied from 188 (fosmid 22G3) to 355 (fosmid 101F8). Comparisons of these indicated similarities in sequence composition between fosmids 22G3, 28C5 and 53D1, next to potential regions of horizontal gene transfer. In particular, the similarities between the latter two fosmids, with several regions showing >40 % similarity (TNC metrics), was remarkable. Subsequent BLASTn analyses of the individual predicted genes as well as the whole-insert sequences suggested all inserts to be derived from bacteria.

**Fig. 1:**
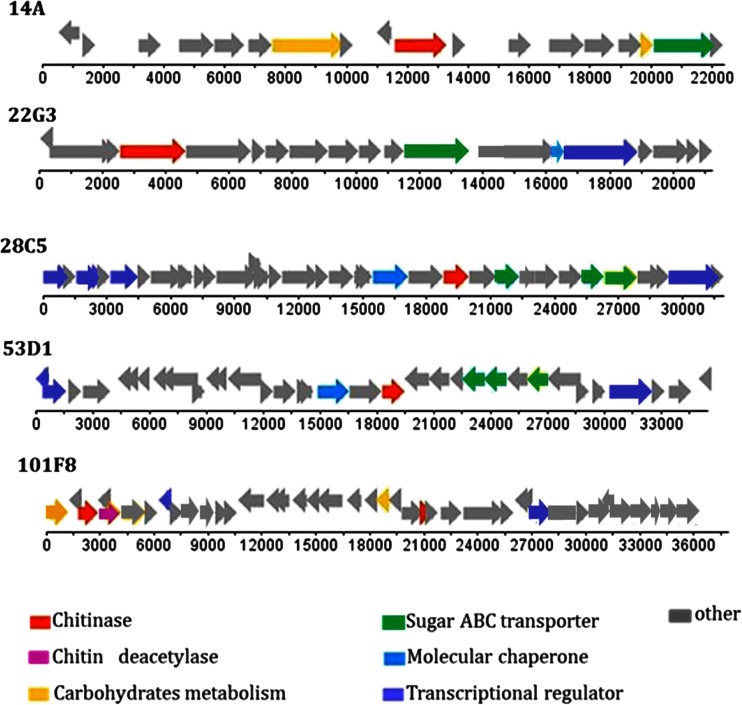
Selected fosmids containing ORFs for predicted chitinolytic proteins. ORF orientation and position of selected genes are shown

With respect to annotation, ORFs for *N*-acetyl-glucosamine transport, sugar ATP-binding cassette (ABC) transport, “molecular chaperoning,” acetylation, transcriptional regulation and (overall) carbohydrate metabolism were consistently, yet variably, present in all fosmid inserts (Fig. 1). Furthermore, tRNA or rRNA genes were not found on any of the fosmid inserts. The full range of predicted functions can be found in Table S3 (A through E). A considerable fraction of CDSs was predicted to be involved in housekeeping and cell replication functions, whereas another fraction was denoted as hypothetical proteins (55–76 %). Table 1 provides an account of all fosmid inserts.

**Table 1:** Distribution of genes for selected proteins and groups of cellular functions among fosmids

Fosmid	Chitinolytic enzyme	Chitin deacetylase	Carbohydrate metabolism^a^	Hydrolase^b^	Transferase	Aldolase	Oxidoreductase	Dehydrogenase	Decarboxylase	ABC sugar –transporter^c^	Efflux transporters	Chemotaxis transducer	Transcriptional regulator	DNA replication	Periplasmic nucleotide binding protein	Membrane protein	Chaperonin	DNA repair	Non-ribosomal synthetase	Antibiotic resistance/synthesis	Plasmid-partitioning protein	Transposase/integrase	Hypothetical	Unknown
14A	1	0	2	0	0	0	0	0	0	1	2	0	0	2	0	0	0	0	0	0	3	1	6	0
22G3	1	0	0	0	1	0	0	0	0	1	2	0	1	0	0	2	1	1	1	0	0	0	7	1
28C5	1	0	0	1	1	1	0	1	0	3	2	1	4	1	1	1	1	0	0	0	0	1	14	0
53D1	1	0	0	2	0	0	0	2	0	3	2	0	3	1	2	1	1	0	1	1	0	2	11	1
101 F8	2	1	2	1	3	0	1	0	1	0	6	0	2	3	1	3	0	0	3	2	0	0	9	0

^a^Other than genes forchitinolytic enzymes and chitinolytic enzyme like sequences

^b^Other than genes for carbohydrate hydrolase

^c^ *N*-acetyl-d-glucosamine ABC transport system and ABC-type sugar transport component

Taking into account the genes related to chitin degradation, carbohydrate metabolism, transport of *N*-acetyl-glucosamine and other sugar-like molecules, transcriptional regulation and chaperoning, the numbers of genes presumably involved in “chitin transformation and general carbohydrate/sugar transport/capture and metabolism” were found to range from 4 (14A) to 9 (28C5) per fosmid. Thus key genomic regions that encode systems involved in chitin/carbohydrate utilization were obtained from as-yet-unknown soil bacteria. In the next section, we describe the salient features of each of the five fosmids.

### Fosmid annotation and prediction of source organism

#### Fosmid 14A

Eighteen ORFs were identified in the insert in the 22.6 kb fosmid denoted 14A (Table S3A). The overall G + C content was 52.7 %. Three intergenic regions were identified. The majority of ORFs revealed a “positive” transcription frame (Fig. 1(14A)). ORF lengths varied from 189 (CDS8 and CDS18, hypothetical proteins) to 2,394 bp (CDS7, closest hit β-d-galactosidase; CAZy GH family 2). One CDS (CDS10, 1,698 bp) was annotated as a putative endochitinase gene with best BLAST hit (99 % similarity, 99 % coverage) to a recently described *K. setae* chitinolytic enzyme (Ichikawa et al. 2010), whereas hits with a similar gene present in any of several *Burkholderia* sp. were also found. The number of hits with genes for carbohydrate metabolism and sugar ABC transport was low. Surprisingly, five CDSs were predicted to encode proteins involved in plasmid partitioning and replication, whereas one phage-type integrase was detected. Another six CDSs remained hypothetical. Nine of the 18 CDSs were affiliated to sequences from a *Burkholderia*-like source organism, at a (protein-based) level of similarity between 27 (CDS6, ABC transporter) and 82 % (CDS14, hypothetical protein) (Table S3A). A *Burkholderia* sp. origin of the fosmid was thus predicted.

#### Fosmid 22G3

Nineteen putative ORFs, with one gap, were identified in the 21.2 kb fosmid 22G3. The overall G + C content was 58.8 %. Only one ORF had a “negative” transcription frame (Fig. 1(22G3); Table S3B). Most CDSs were large, with 63 % being >0.5 kb. One CDS typical for a chitinolytic enzyme was found. It was identical to a gene for chitinolytic enzyme “A” of *Acidobacterium capsulatum* ATCC 51196. Another CDS, encoding an *N*-acetyl-glucosamine transporter, was affiliated to a similar CDS of the same strain (similarity 51 %, coverage 86 %). Moreover, a transcriptional regulator (similarity 33 %, coverage 78 %), which was similar to a region from *Granullicella tundricola* MP5ACTX9, was found downstream of the aforementioned gene. One CDS for a chaperonin-like protein (universal stress protein-related) and one for a sugar ABC transporter was found (Table 1). Eleven of the 19 CDSs were similar to genes from acidobacteria resembling *A. capsulatum*, making this the most likely source organism (Table S2B).

#### Fosmid 28C5

A contiguous sequence, containing 34 ORFs with positive transcription frame, was assigned for the 31.9-kb fosmid 28C5 (Fig. 1(28C5); Table S3C). The average G + C content was 65.2 %. The sizes of the CDSs ranged from 144 (CDS9—hypothetical short protein) to 2,310 bp (CDS33- transcriptional regulator). One CDS (CDS23, −1,188 bp) was annotated as an ORF predicted to encode a chitinolytic enzyme (best BLAST hit to agene encoding a chitin-active protein from *S. maltophilia* AU12-09; 45 % similarity, 88 % coverage). Another CDS downstream of the former one was predicted to encode an *N*-acetyl-glucosamine ABC transporter (best hit—25 % similarity, 85 % coverage—to a *Streptomyces bingchenggensis* BCW-1 transporter). Other putative ORFs, such as those for transcriptional regulators and chaperonin GroEL, were also found. These were affiliated, at similarity levels ~38 % and high coverage (98 %), to similar sequences from *Chloroflexus*-like organisms, in particular *N. hollandicus* and *K. racemifer.* One duplicated gene was assigned to a gene encoding a hypothetical protein from *N. hollandicus* Lb (similarity 44 %, coverage 73 %). Overall, the analyses suggested an organism belonging to the *Chloroflexi*, affiliated with *K. racemifer* and/or *N. hollandicus,* to be the most likely source organism.

#### Fosmid 53D1

A total of 34 ORFs with positive and negative transcription frames were identified in the 35.4 kb fosmid 53D1 insert sequence (Fig. 1(53D1), Table S3D; one gap). The overall G + C content was 54.6 %. The CDS sizes ranged from 144 bp (CDS6, truncated part affiliated with transposase IS66) to 2,310 bp (CDS31, putative protein kinase /transcriptional regulator). The 144-bp element similar to IS66 was puzzling. Genes for hypothetical proteins represented 32 % of the 53D1 sequence. One CDS for a chitinolytic enzyme (CDS20, 1,191 bp) was identified. The best BLAST hit of this CDS was with a gene from an “uncultured bacterium” (48 % similarity, 94 % coverage), followed by one with an *S. maltophilia* AU12-09 gene (45 % similarity, 87 % coverage) and a *K. racemifer* DSM 44963 one (41 % similarity, 93 % coverage; resembling a gene from *N. hollandicus)*. Moreover, *N*-acetyl-glucosamine transporter and sugar ABC transporter genes, affiliated with genes from *K. racemifer* DSM 44963, occurred downstream of CDS20. Similarly, for the CDSs corresponding to transcriptional regulators and to hypothetical proteins flanking the gene, a *Chloroflexus*-type source organism was predicted (Table S3D). Overall, of the CDSs annotated as *Chloroflexus-*associated genes, 35 % had as close homologs genes from the recently described *N. hollandicus,* which is a close relative of *K. racemifer* (Sorokin et al. 2012).

#### Fosmid 101F8

Fosmid 101F8 was found to contain a 37,907-bp insert. In total, 40 CDSs were found, with positive and negative transcription frames (Fig. 1(101F8); Table S3E). The CDS sizes varied from 231 (CDS4, hypothetical protein), to 1,581 bp (CDS33, putative sensory transduction protein), to 2,316 (CDS28, predicted penicillin-binding protein). The G + C content was 59.5 %. Two different CDSs for putative chitinolytic enzymes, next to one putative chitin deacetylase gene were found. CDS3 (1,122 bp; best BLAST hit—100 % identity and coverage—to a region from *N. koreensis* GR20-10) and CDS25 (405 bp; best BLAST hit—77 % similarity, 82 % coverage—to *Streptomyces avermitilis* MA-4680) were annotated as putative chitinase-encoding genes. CDS5 (870 bp), being identical to a genomic region of *Thermodesulfatator indicus* DSM15286, was assigned as belonging to a gene family encoding polysaccharide deacetylase proteins. With the exception of CDSs for the chitinolytic enzymes, the putative deacetylase and one antiporter protein (CDS26), all CDSs were affiliated, with high similarity and coverage values, to genes of *Aeromonas* spp., with 72 % of these CDS being related to *Aeromonas veronii* genes (Table S3E).

### Genes and regions of similarity between the fosmids

**Fig. 2:**
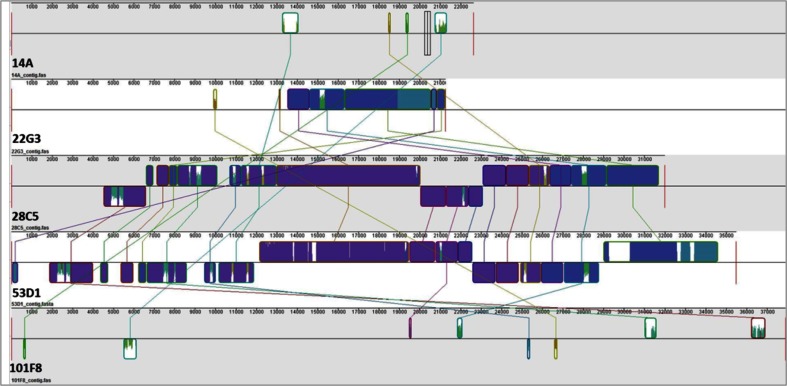
Comparison of full-length fosmid insert nucleotide sequences using progressive Mauve global and local alignment. Progressive Mauve yields an ungapped local multiple alignment with components from input sequences. The panels show: (1) sequence coordinates of the fosmid, (2) colored block outlines above and below center line; *above*: forward orientation, *below*: reverse orientation (complement), (3) each block outline surrounding a fosmid region with homology to a region from another fosmid, internally free from rearrangements, (4) *regions outside blocks*: no detectable homology among fosmids, (5) *inside blocks*: similarity profiles of sequences; height: average level of conservation, (6) *white*: region not aligned, probably specific to a particular genome, (7) *connecting lines*, indicating ungapped regions that are homologous. *Different colors*: different block-specific sequences

Although the genomic organizations were unique per fosmid insert, several common features were identified between them. All fosmids contained transcriptional regulators of the *LuxR*, *LitR* and *LysR* types, as well as sugar ABC transporter genes. Fosmids 22G3 and 101F8 revealed the presence of CDSs predicted to encode a putative chitinolytic enzyme close to the 5′-ends of the insert. Fosmids 22G3, 28C5 and 53D1 presented, downstream from the ORF for the putative GH18 chitinase, one copy of an ORF encoding a protein potentially involved in *N*-acetyl-glucosamine transport. Nucleotide frequency analyses indicated the presence of overlapping values between fosmids 28C5 and 53D1 (40.3 % similarity), and to a lesser extent 28C5 and 22G3 (12.1 % similarity) and 14A and 22G3 (7.3 %). Progressive Mauve alignment of the ORF nucleic acid sequences showed the existence of 13 regions of significant similarity between fosmids 28C5 and 53D1 (Fig. 2), the localization of which appeared, however, scrambled.

### In silico analysis and selection of a candidate gene for enzyme expression and characterization studies

**Fig. 3:**
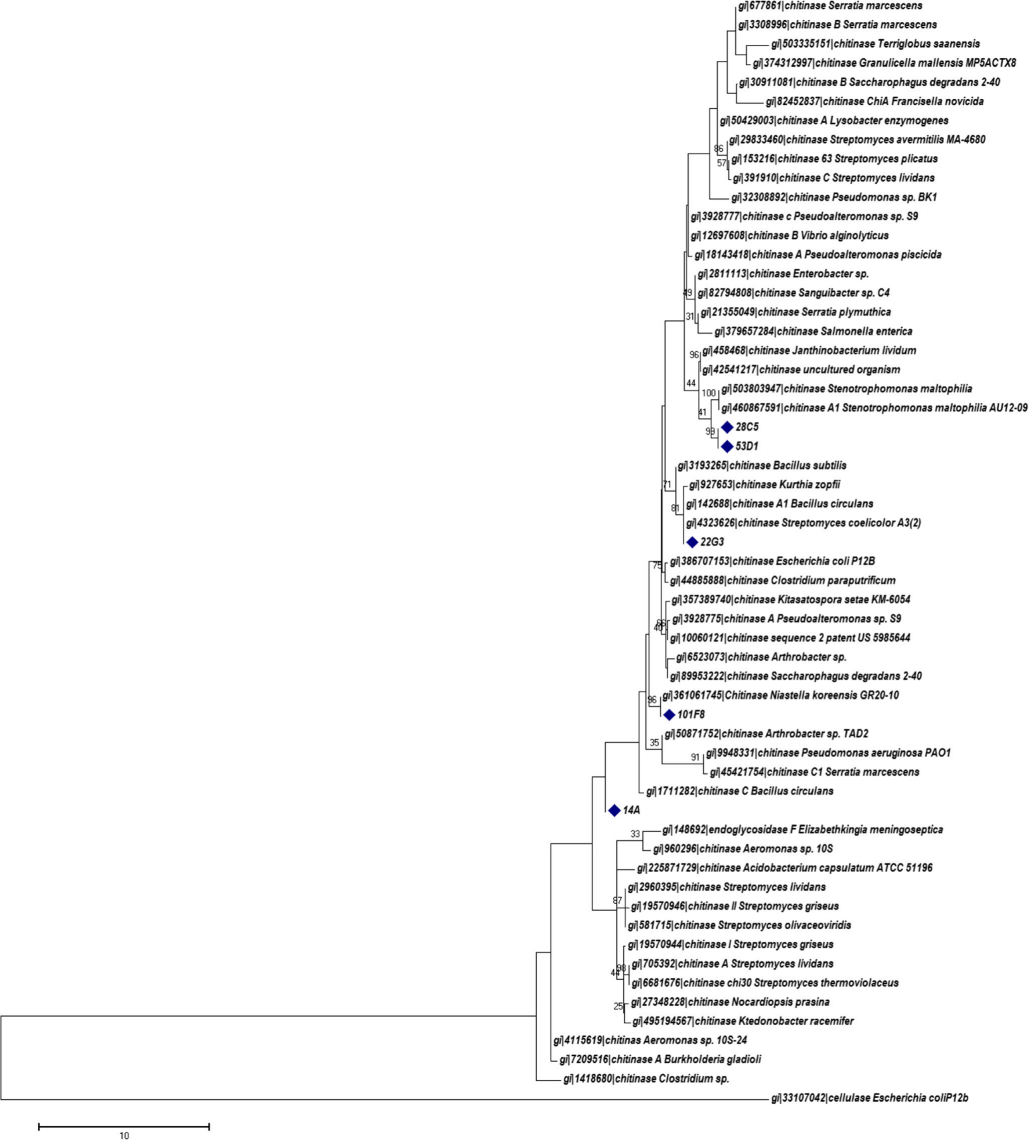
Maximum likelihood phylogenetic analysis of chitinolytic enzyme sequences obtained in this study (*marked*) and 60 sequences of representative chitinolytic enzymes retrieved from the CAZy data base. Substitution model of Jones–Taylor–Thorton, uniform rates, partial deletion, and site coverage cutoff 95 %. Bootstrap values >25 are indicated

All fosmid inserts were confirmed to be part of predicted chitinolytic enzyme-encoding gene clusters. We first decided to check for nearest relatives/neighbors in databases using phylogenetic analyses of the genes for the putative chitinolytic enzymes (based on predicted protein sequences). These analyses showed clustering with several reference chitinolytic enzymes retrieved from the CAZy database and distant from the outgroup sequence of *E. coli* P12b cellulase (Fig. 3). The close affiliations with genes from organisms related to *S. maltophilia* (fosmids 28C1 and 53D1), *N. koreensis* GR20-10, *S. coelicolor* A3(2) and *K. setae* KM-6054 were confirmed (Fig. 3).

**Fig. 4:**
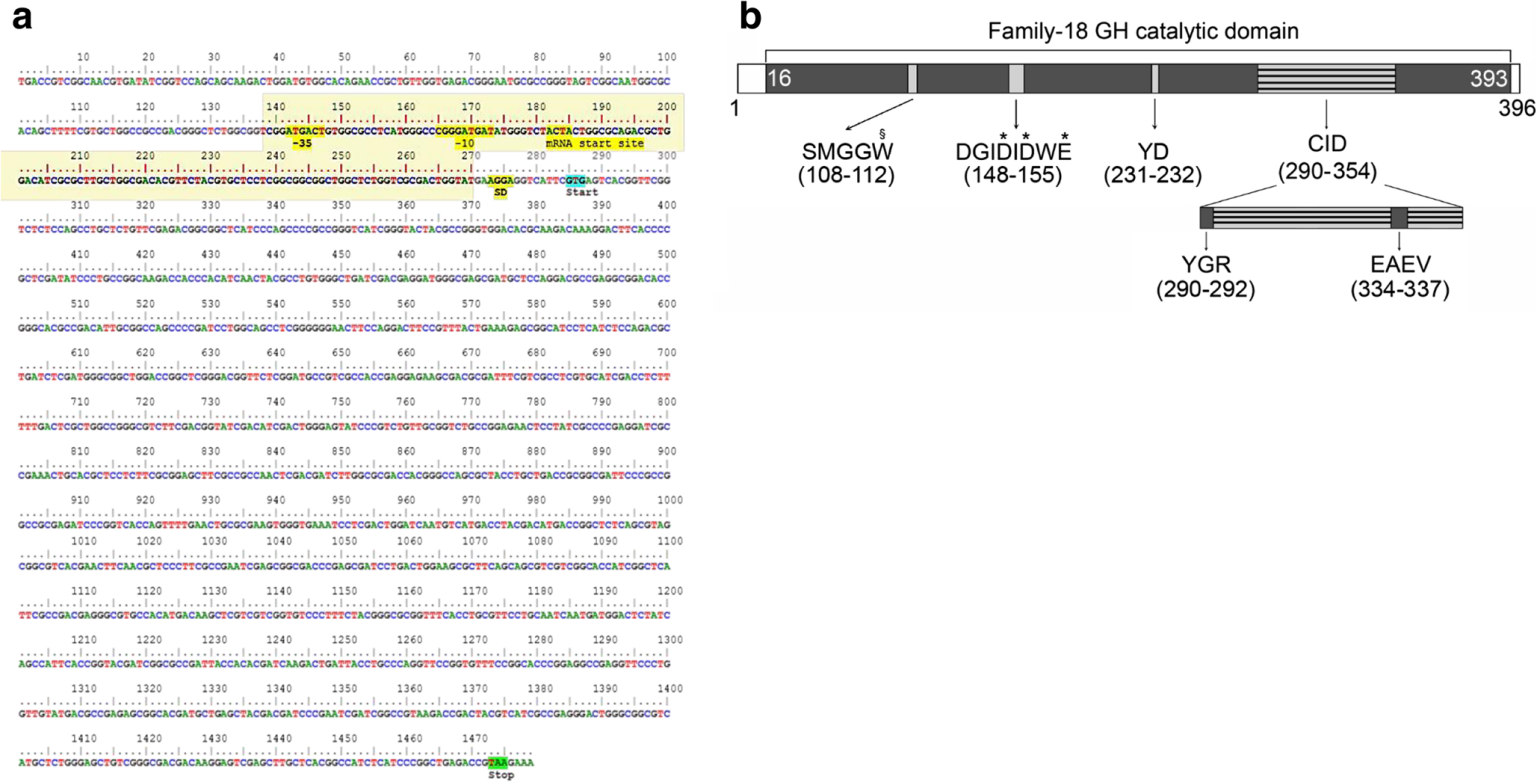
Analysis of the 53D1 *chi*A-like ORF and encoded protein. **a** DNA level. Predicted RNA polymerase interaction region (*shadowed*), −35 and −10 regions, mRNA start site, Shine–Dalgarno region, start, and stop codon are *marked*. The presence of these elements was inferred by using BProm (SoftBerry) and the RBS Calculator. **b** Protein level. Motifs identified within the 396-amino-acid long 53D1 polypeptide chain include a family-18 GH catalytic domain (amino acids 16–393) and a chitin insertion domain (CID, 290–394). The conserved consensus sequences within the catalytic domain and the CID are *highlighted*, with the corresponding amino acid positions. Conserved residues essential for catalysis are indicated by *asterisks*; the Trp presumably involved in the processivity of the chitinase is indicated by *section sign*. Sequence analysis was performed using InterPro Scan and Prosite

Fosmid 53D1 was selected for further analyses. Given the fact that the *chi*A-gene forward primer used anneals with the [D/N]G[L/I/V/M/F][D/N][L/IV/M/F][D/N]xE consensus sequence of 53D1 inside the catalytic domain and the reverse primer with the first CID sequence inside the catalytic domain, we predicted the 53D1 sequence to encode a chitinase belonging to subfamily A of family GH-18, as reported by Williamson et al. (2000). Based on the annotation of the ORF for the predicted chitinolytic enzyme, we closely examined the coding region (63.03 % G + C) together with 200 nucleotides upstream of the identified start codon GTG (encoding Val). Thus, essential genetic elements (promoter, RNA polymerase interaction site, Shine–Dalgarno sequence, start and stop codons) necessary for expression in *E. coli* were identified (Fig. 4a). The 53D1 ORF falls into the 14 % of bacterial genes with an unusual promoter region and codon start (Tikole and Sankararamakrishnan 2006; Nakamoto 2009). The “-35 to -10” region revealed the atypical sequence “ATGACT…CGGGAT,” while the Shine–Dalgarno sequence was the universal AGGAGGT.

The predicted protein was 396 amino acids long (having a 43.6 kDa estimated molecular mass and a theoretical pI of 4.83). Its domain organization, as predicted by InterPro Scan and Prosite analysis, is shown in Fig. 4b. About 95 % of the amino acid sequence is composed of a catalytic domain (positions 16–393), which shows the highest sequence identity with the catalytic domains of the family-18, subfamily A chitinases from *Stenotrophomonas maltophila* (46 % identity), *Pseudomonas geniculata* (44 %), *Amycolatopsis nigriscens* (44 %), and *Desmospora* sp. 8437 (42 %).

The 53D1 catalytic domain revealed the presence of three consensus sequences that are highly conserved within family-18 GHs, i.e., DGIDIDWE (residues 148–155, corresponding to the consensus motif [D/N]G[L/I/V/M/F][D/N][L/IV/M/F][D/N]xE, PROSITE accession number PS01095), YD (position 231–232, consensus Y[D/N]) and SMGG (residues 108–111, consensus SxGG) (Vaaje-Kolstad et al. 2013; Watanabe et al. 1993). The first region contains the highly conserved proton donor glutamic acid residue (Glu155), and in addition two aspartic acid residues (Asp151 and Asp153), which are frequently reported in family-18 GHs and are considered to be equally important for the hydrolytic mechanism: Asp153 is supposed to contribute to the correct orientation of the *N*-acetyl group of the substrate *N*-acetyl glucosamine and to the stabilization of the oxazolinium ion intermediate, and Asp151 to the protonation of the other aspartic acid residue (Lobo et al. 2013; Vaaje-Kolstad et al. 2013). Interestingly, the SMGG motif of 53D1 is followed by a Trp (Trp112), this association being considered vital for the processive behavior of *S. marcescens* ChiA and ChiB (Payne et al. 2012).

Analysis of the 53D1 sequence also confirmed the presence of a 64-residue CID within the catalytic domain (positions 290–354), which is a distinctive feature of subfamily A of the family-18 GHs. As highlighted in Fig. 4b, the 53D1 sequence contains the two conserved sequences of CIDs, the YxR motif in the N-terminal region (YGR, positions 290–292) and the [E/D]xx[V/I] motif in the center (EAEV, 334–337). Differently from other bacterial chitinases, the 53D1 sequence does not contain other auxiliary modules, such as CBDs or FnIII domains (Suzuki et al. 1999; Li and Greene 2010; Eijsink et al. 2010). In addition, no putative signal peptide was identified at the N-terminal of the protein, neither for the Sec nor for the TAT secretion pathways (Francetic et al. 2000a, b; Lobo et al. 2013). Manual alignment of the 53D1 sequence with proteins secreted through the type IX secretion system (T9SS) did not identify any similarity with the conserved C-terminal domain (CTD) of 60–100 amino acids that target these for secretion (Kharade and McBride 2015). Recently, it has been reported that a novel ChiA is secreted by T9SS in the gliding bacterium *F. johnsoniae* (Kharade and McBride 2014).

### Expression, purification and characterization of the 53D1 chitinolytic enzyme

The amplified *53D1* gene region was cloned into pET24b(+) in *E. coli* BL21 Star^TM^(DE3) under the influence of the IPTG-inducible *T7* promoter. In addition, we used the pColdI system, which is based on low-temperature expression to improve the solubility of heterologous proteins (Qing et al. 2004). In both systems, most of the recombinant protein (>80 %) accumulated in insoluble fractions, being up to 20 % detectable in the soluble fraction (Supplementary Figs. S1, S2 and S3). Fluorimetric enzyme activity assays revealed that the cytoplasmic protein was active, at ca. 6 U/g cells, in the pET24b(+) clones (Fig. S2), whereas the accumulated insoluble form appeared as mostly inactive. ChiA protein was not detected when the *53D1* gene was cloned under the control of its native promoter (neither by chitinolytic enzyme assay nor by immunoblotting).

**Fig. 5:**
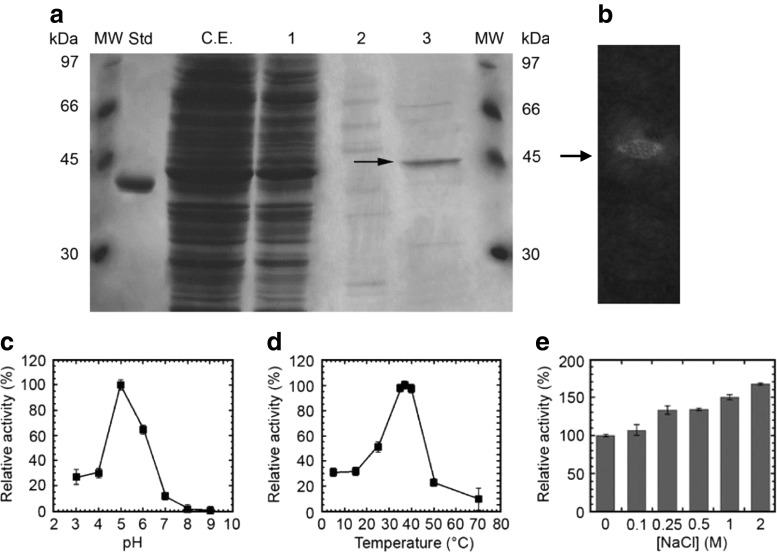
Detection of the 53D1-encoded chitinolytic protein from *E. coli* BL21 Star^TM^(DE3)/pET24b(+)::*53D1* cells.** a** SDS-PAGE analysis of chromatography fractions. *C.E.*: crude extract; *1*: flow-through; *2* and *3*: fractions eluted at 125 and 500 mM imidazole, respectively. *Std*: standard reference protein, d-amino acid oxidase from *Rhodotorula gracilis* (42.5 kDa, 10 μg) gently provided by Loredano Pollegioni, University of Insubria, Italy. *MW*: molecular weight markers, from GE Healthcare Sciences, Little Chalfont, UK. 53D1 protein spot indicated by *arrow*. **b** Zymogram analysis in semi-native conditions of the purified 53D1 with carboxymethyl-chitin-remazol brilliant violet as substrate. 53D1 protein spot, visualized as a chitin degradation halo, indicated by *arrow*. Effect of pH (**c**), temperature (**d**), and increasing concentrations of NaCl (**e**) on chitobiosidase activity of the purified 53D1 protein, using 4-MU-(GlcNAc)_2_ as the substrate. *Enzyme activities are reported as relative to the activity* of 45.2 U/mg protein (set as 100 %) measured at pH 5, 37 °C and in the absence of salt. *Values represent the means of three independent experiments (mean ± standard error)*

Following purification, SDS-PAGE analysis showed that the 53D1-derived protein migrated as a single band of 44.7 kDa (expected molecular mass for the recombinant His_6_-tagged protein) and was >80 % pure (Fig. 5a). Zymograms on carboxymethyl chitin confirmed the activity of the enzyme on chitin (Fig. 5b). The protein showed prevalent chitobiosidase activity on 4-MUF-(GlcNAc)_2_ (45.2 U/mg), weaker endochitinase activity on 4-MU-(GlcNAc)_3_ (21.2 U/mg) and no β-*N*-acetyl-glucosaminidase activity on 4-MU-GlcNAc. The enzyme was also able to hydrolyze colloidal chitin, with an activity of about 2.3 U/mg.

Using the purified protein, its chitobiosidase activity was then assayed over a pH range of 3.0–9.0 and a temperature range of 5–70 °C. The optimum pH of protein 53D1 was 5.0; >60 and 30 % of the activity were maintained at pH 6.0 and 3.0–4.0, respectively (Fig. 5c). At pH >6.0, the activity decreased drastically. The optimum temperature of function was between 35 and 40 °C. However, more than 30 % of the activity was retained even below 15 °C, and more than 20 and 10 % at 50 and 70 °C, respectively (Fig. 5d).

The effects of several compounds on chitobiosidase activity were then evaluated (Table 2). Among the metal ions tested, Mg^2+^ and Co^2+^, as well as NH_4_^+^, did not significantly affect the hydrolytic activity of the 53D1 protein, while Cu^2+^, Fe^2+^, Mn^2+^, and Zn^2+^ reduced it, with the strongest inhibition being due to Cu^2+^ and Fe^2+^. In contrast, Ca^2+^, K^+^, and Ni^2+^ slightly increased the activity of the enzyme. Incubating the enzyme with the chelating agent EDTA inhibited chitobiosidase activity, suggesting that metal ions are needed for 53D1 maximum catalytic activity.

**Table 2:** Characteristics of fosmid 53D1-derived chitobiosidase

Compounds	Final concentration	Relative activity (%)
Control		100
Metal ions	mM	
Ca^2+^	20	126 ± 4.3
Cu^2+^	20	7.6 ± 0.1
Fe^2+^	20	15.9 ± 1.9
K^+^	20	116 ± 0.6
Mg^2+^	20	98.0 ± 1.3
Mn^2+^	20	65.8 ± 2.5
Ni^2+^	20	132 ± 1.1
NH_4_^+^	20	99.8 ± 2.9
Zn^2+^	20	30.3 ± 2.0
Co^2+^	20	90.4 ± 0.9
EDTA	20	65.2 ± 1.3
Enzyme inhibitors	% (*v/v*)	
β-Mercaptoethanol	5	10.3 ± 6.3
DTT	5	11.8 ± 4.4
Detergents	% (*w/v*)	
SDS	1	0.65 ± 0.1
Triton X-100	1	119 ± 1.8
Tween-20	1	116 ± 2.6
DOC	1	40.6 ± 4.9
Nonidet P-40	1	115 ± 1.4
NLS	1	76.6 ± 1.8
Sugars	mM	
NAG	10	120 ± 3.3
Chitobiose	10	92.0 ± 1.4
Organic solvents	% (*v/v*)	
Ethanol	10	48.9 ± 2.6
Methanol	10	64.8 ± 3.9
Isopropanol	10	61.0 ± 0.5
DMSO	10	50.9 ± 4.4

The enzyme activity is expressed relative to the activity of 45.2 U/mg protein (set as 100 %) on 4-MU-(GlcNAc)_2_ as the substrate, at 37 °C in 100 mM sodium acetate (pH 5.0)

The enzyme inhibitors β-mercaptoethanol and DTT strongly reduced enzyme activity. The influence of a variety of detergents on 53D1 is shown in Table 2. SDS, DOC, and NLS showed inhibitory effects, while other detergents (Triton X-100, Tween-20 and Nonidet P-40) had no effect or even slightly increased the chitobiosidase activity. The functional stability of 53D1 was then evaluated in a panel of organic solvents. All solvents, i.e. ethanol, methanol, isopropanol and DMSO, significantly reduced the activity, with an average residual activity in the range 45–65 %. Furthermore, activity was slightly inhibited in the presence of 10 mM chitobiose and slightly increased in the presence of 10 mM *N-*acetyl-d-glucosamine. Very interestingly, the 53D1 enzyme was resistant to, or even dependent on, high salt concentrations, as its catalytic activity increased in the presence of NaCl, up to a 2 M final concentration (Fig. 5e). Analyzing the amino acid composition of 53D1 according to Bolhuis et al. (2008), the chitobiosidase showed features of halophilic organism-derived proteins, including: (1) an increased number of acidic residues (Glu, Asp—14.4 % in 53D1, vs the average value of 10.9 % in *E. coli* proteome), of Ser and Thr (14.4 vs 11.1 %), and of small hydrophobic residues (Ala, Gly and Val—29.6 vs 23.7 %); (2) a decreased number of Lys (2.5 vs 4.7 %) and of large hydrophobic residues (Ile, Leu, Phe and Met—18.9 % *vs* 23.5 %) (Bolhuis et al. 2008; Reed et al. 2013; Oren and Mana 2002). That 53D1 chitobiosidase has features of a protein with hypersaline adaptation was confirmed by applying the models (principal components analysis, PCA; partial least squares regression, PLSR; linear regression, LR) developed by Zhang and Ge (2013). The abundance of acidic amino acids, the prevalence of Asp and His (abundant in halophilic proteins), in contrast to Phe and Ile (scarce in halophilic proteins), and finally the low content of Cys, Ile and Gly vs the high content of Asp, His, Gln, Glu and Arg confirmed that the 53D1 chitobiosidase is a halophilic protein.

## Discussion

Considering the prevalence of prokaryotic organisms in soil and their average genome sizes (estimated to be about 5 Mb; Hardeman and Sjoling 2007), the fosmid library produced from the chitin-amended field soil represented microbial community DNA equivalent to approximately 1,200 genomes. Genetic screening of this metagenome from the chitin-amended soil had, as the main objective, the identification of genes for novel proteins that belong to the functional group of chtinolytic enzymes. The screening strategy applied was based on the use of the *chi*A-gene based degenerated primers as the proxy for the genetic basis of chitinolytic enzyme activity. One may argue that this screening strategy is contentious; however, it allowed us to screen the huge sequence space (Kielak et al. 2013) around the *chi*A genes that are currently known. The strategy proved to be successful in recovering several genomic fragments containing novel ORFs for putatively active chitinolytic enzymes. In total, four sequences that encode such novel enzymes were found in five fosmids (one found twice, in different genetic contexts), next to a considerable number of (flanking) sequences related to the metabolic pathway of carbohydrate degradation, transport and excretion, next to the regulation of transcription. Given the estimated proportion of *chi*A-related genes in soil bacteria (roughly 1–5 % of a random representative sample of bacterial cells from soil), the frequency of recovery of *chi*A*-*positive clones was consistent with the estimated frequency, and corroborated that found in other reports (Wellington et al., *in preparation*).

The origins and source organisms of the genes for the putative chitinolytic proteins were found to be diverse, indicating that these genes were spread across diverse dominating organisms in the chitin-amended field soil. Overall, the majority of the predicted genes on the fosmids revealed homologies to regions of the genomes of organisms like *Burkholderia*, *Acidobacterium*, *Aeromonas* and two related *Chloroflexi*. The latter fosmids, 28C5 and 53D1, comprised genes which were similar to those described in the chloroflexi *K. racemifer* and *N. hollandicus* (Sorokin et al. 2012), whereas the *chi*A-like gene on both fosmids was identical. The identification of a *Chloroflexus*-like chitinase gene (although also related to a similar gene from *Stenotrophomonas*) in a metagenome from a chitin-enriched habitat may indicate another asset of the remarkable physiology of *N. hollandicus*-like organisms. In fact, *N. hollandicus* has been described as a nitrite oxidizer, being the only organism of this guild that is not affiliated to the *Proteobacteria*.

The bioinformatics analysis of the selected 53D1 sequence predicted it to have chitinolytic activity (similar to the one present on fosmid 28C5). The analysis suggested it is a chitinase belonging to subfamily A of family-18 GH, composed for 95 % by the catalytic domain containing the characteristic CID motif that provides a “wall” of the substrate binding cleft. This indicated that the enzyme might act in a processive way (also called “multi attack”). No other auxiliary modules, such as CBDs, FnIII domains or peptide signals, were present. The features of the 53D1 ChiA protein (and the gene encoding it) were remarkable. First, the gene was not expressed in *E. coli* from its own promoter, which may relate to the failure to establish ‘expression conditions’. Interestingly, it is known that *chi*A genes can remain cryptic under standard laboratory conditions (Francetic et al. 2000a, b). The protein expressed from vector-based expression signals showed features that characterize it as a typical “temperate-climate-soil” enzyme, as it was active under moderate temperature and pH conditions, which reign in many soils in temperate climate zones. Second, it was sensitive to organic solvents, indicating it was not selected to withstand selective pressure from such sources. Remarkably, protein 53D1 showed tolerance to elevated levels of NaCl, increasing its activity at the higher salt levels, being halophilic rather than halotolerant. The halophilic behavior of the enzyme was confirmed by the analysis of its amino acid composition; like other halophilic proteins studied in Archaea and in some bacteria, the 53D1 protein has an increased content of acidic amino acid residues and a decreased hydrophobicity (Reed et al. 2013; Oren and Mana 2002). A few halotolerant bacterial chitinases have been hitherto characterized, key examples being described by Konagaya et al. (2006) and LeClair et al. (2007). However, only two chitinases (type C, Chi-I and Chi-II; from the halophilic bacterium *Salinivibrio costicola*, Aunpad et al*.*^2007^) so far showed a behavior similar to the 53D1 enzyme, with a salinity optimum at 1–2 % NaCl, a residual activity of more than 80 and 50 % in the presence of 3–5 and 14 % NaCl, respectively, and 95 % activity without salt addition. Such halophilicity is more common among archaeal chitinases, with some of them being active in the absence of salt but showing maximum activity in high-salt conditions (Garcia-Fraga et al. 2014). Others are not only adapted to high concentrations of salt, but also need a variable amount of NaCl for correct folding (Gomes and Steiner 2004; Litchfield 2011). The salt tolerance of the 53D1 chitin-active enzyme was thus very interesting, as it (1) points to an in-situ enzymatic activity of which the level may depend on the presence of salt, and (2) may play a role in soil microhabitats where salt accumulates, i.e., in soils under increasing salt stress resulting from evaporating water.

Fourth—and biotechnologically relevant—the 53D1-encoded protein was typified as a chitobiosidase, which is active on colloidal chitin and not only on the chito-oligosaccharide analogues that are commonly used for chitinolytic enzyme detection. Consistently, as argued above, the catalytic domain of the 53D1 chitinase contains the conserved CID domain, which structural studies on other family-18 chitinases revealed to be involved in binding and processing long-chain substrates like chitin (Li and Greene 2010; Zees et al. 2009). These aspects, in combination with the remarkable salt tolerance of the 53D1 ChiA protein, make it an interesting candidate for the treatment of seafood waste such as shrimp carapace.

Finally, the fact that the 53D1 enzyme may have derived from a bacterium related to the *Chloroflexus* species *K. racemifer* or *N. hollandicus,* next to its occurrence on fosmid 28C5, indicated that as-yet-uncultured organisms that are affiliated with the mentioned *Chloroflexus* types may play important roles in soils in which a substrate like chitin (that feeds them with respect to their carbon as well as nitrogen needs) is prevalent. Possibly, their value for the inferred source bacteria lies in their potential chito oligosaccharide cleaving activity under drought stress, which comes with enhanced levels of dissolved salts in the soil solution. Furthermore, one may surmise that, in their ecological functioning, these organisms, being parts of complex communities “in action” on the substrate offered, may be involved in ample recombination/genome reshuffling processes, giving rise to diverse forms. The remarkable differences in the genetic backgrounds of the *chi*A-type gene on fosmids 53D1 and 28C5 indeed indicate the occurrence of recombinations involving the regions surrounding the gene.

## Electronic supplementary material

ESM 1 (PDF 370 kb)
