# Supplementary material for: A novel salt-tolerant chitobiosidase discovered by genetic screening of a metagenomic library derived from chitin-amended agricultural soil
Source: Appl Microbiol Biotechnol. 2015 Jun 4;99(19):8199–215. doi: 10.1007/s00253-015-6639-5 (PMC4561078; doi:10.1007/s00253-015-6639-5)
Supplement: Supplementary file 1 — (PDF 370 kb) [file 253_2015_6639_MOESM1_ESM.pdf]

Supplementary material

Applied Microbiology and Biotechnology

A novel salt-tolerant chitobiosidase discovered by genetic screening of a metagenomic library derived from chitin-amended agricultural soil

Mariana Silvia Cretoiu[1,2], Francesca Berini[3], Anna Maria Kielak[4], Flavia Marinelli[3], Jan Dirk van Elsas[1]

[1]Department of Microbial Ecology, CEES, University of Groningen Groningen, The Netherlands
[2]Department of Marine Microbiology, Royal Netherlands Institute for Sea Research, Yerseke, The Netherlands
[3]Department of Biotechnology and Life Sciences, University of Insubria, and "The Protein Factory" Research Center, Politecnico of Milano, ICRM CNR Milano and University of Insubria, Varese, Italy
[4]Department of Microbial Ecology, The Netherlands Institute of Ecology (NIOO), Wageningen, The Netherlands

Corresponding author: Jan Dirk van Elsas (j.d.van.elsas@rug.nl)

Supplementary figures

Figure S1: Western blot analysis of *E. coli* BL21 Star^{TM}(DE3) cells carrying pET24b(+) or pET24b(+)::*53D1* plasmids, grown in LB medium for 2 h at 37°C after induction

Figure S2: 53D1 expression levels in *E. coli* BL21 Star^{TM}(DE3)/pET24b(+)::*53D1* recombinant strain

Figure S3: 53D1 expression levels in *E. coli* BL21 Star^{TM}(DE3)/pCOLDI::*53D1* recombinant strain

Supplementary tables

Table S1: Fosmid functional annotation criteria

Table S2: Fosmid characteristics

Table S3: Complete annotation of five selected fosmids carrying *chi*A-like genes
A. 14A; B. 22G3; C. 28C5; D. 53D1; E. 101F8

53D1 chitinase gene expression

Materials and Methods

Protein expression experiments were conducted in Luria Bertani (LB) and Terrific Broth (TB), both purchased from Sigma-Aldrich, St Louis, USA. Starter cultures were prepared from a single recombinant *Escherichia coli* colony in 10 ml LB medium grown overnight at 37°C and 200 rpm. Buffled 500 ml Erlenmeyer flasks containing 100 ml of cultivation medium were inoculated with the starter culture (initial OD_{600nm} = 0.1) and further incubated as before. In the case of *E. coli* BL21 Star^{TM}(DE3) cells transformed with either pColdI::*53D1* or pET24b(+)::*53D1* vectors, protein expression was induced adding 0.5 mM IPTG to cells in the early-exponential growth phase (OD_{600nm} ~0.6 in LB, ~1.0 in TB). After induction, *E. coli* BL21 Star^{TM}(DE3)/pColdI::*53D1* cells were cultured at 15°C and 200 rpm according to manufacturer's instructions, while *E. coli* BL21 Star^{TM}(DE3)/pET24b(+)::*53D1* cells were incubated at 37°C or 25°C and 200 rpm. Cells were harvested at regular time intervals by centrifugation (1,900 x *g* for 30 min) at 4°C. Supernatants (i.e., the cell-free fermentation broths) were treated with 10% v/v trichloroacetic acid. Cell pellets were instead sonicated on ice (3-5 cycles of 30 sec each, with a 30-sec interval, using a Branson Sonifier 250, Danbury, USA) in Phosphate Buffer Saline (PBS) pH 7.3 containing 10 µg/ml deoxyribonuclease (DNase, Sigma-Aldrich, St Louis, USA), 0.19 mg/ml phenylmethylsulfonylfluoride (PMSF, Sigma-Aldrich, St Louis, USA) and 0.7 mg/ml pepstatin (Sigma-Aldrich, Str Louis, USA). Soluble and insoluble fractions were then separated by centrifugation (20,000 x *g* for 40 min) at 4°C. Insoluble fractions (containing membrane and inclusion bodies) were re-suspended in a volume of PBS equal to the corresponding cytoplasmic soluble fraction (2-3 ml per gram of cells) for successive analyses. Protein concentration was determined by Biuret assay (Gornall et al. 1949). Chitinase production was estimated through densitometric analysis of SDS gel bands with the software Quantity One (Bio-Rad Laboratories, Hercules, USA) and His_6-glycin oxidase (His_6-GO) from *Bacillus subtilis* gently provided by Loredano Pollegioni, University of Insubria (Job et al. 2002) as a standard. Chitinase activity was measured by the fluorimetric assay on 4-MU-$(GlcNAc)_2$ (Hjort et al. 2014).

Results

Basal expression was performed with *E. coli* BL21 Star^{TM}(DE3)/pET24b(+)::*53D1* in LB cultures, adding 0.5 mM IPTG when OD_{600nm} reached 0.6 and collecting cells after an additional 2 h of growth at 37°C. Using these conditions, most of the recombinant protein, corresponding to a band of 44.7 kDa, accumulated in insoluble fractions, being a low amount of protein detectable in the

soluble fraction (Supplementary Fig. S1). Protein was not found in medium fractions, nor in fractions collected from control *E. coli* BL21 Star^{TM}(DE3) cells carrying the empty vector.

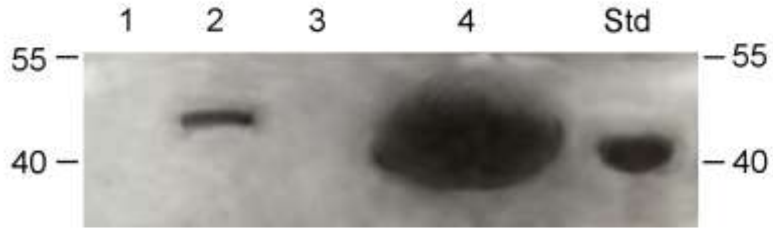

Supplementary Figure S1. Western blot analysis of *E. coli* BL21 Star^{TM}(DE3) cells carrying pET24b(+) or pET24b(+)::*53D1* plasmids, grown in LB medium for 2 h at 37°C after induction. From *E. coli* BL21 Star^{TM}(DE3)/pET24b(+): soluble (lane 1) and insoluble (lane 3) fractions; from *E. coli* BL21 Star^{TM}(DE3)/pET24b(+)::*53D1*: soluble (lane 2) and insoluble (lane 4) fractions. In each lane, samples corresponding to 2 ml of cell culture were loaded. Std reference protein: His_6-GO from *Bacillus subtilis* (10 μg, 42.66 kDa) gently provided by Loredano Pollegioni, University of Insubria (Job et al. 2002).

Chitinase activity assays revealed that the low amount of soluble protein in the above conditions was active (ca. 1 U per g of wet cells), whereas the insoluble form appeared inactive. Supplementary Figures S2a and b report the results from experiments conducted to increase the yield of active soluble 53D1 chitinase. Replacement of LB with the richer TB medium did not increase yield, whereas optimization of the expression conditions (i.e., incubating cells overnight at 25°C after IPTG addition) yielded up to 6 U of activity per g cells, even if >80% remained insoluble and inactive (Supplementary Fig. S2). When *E. coli* BL21 Star^{TM}(DE3)/pColdI::*53D1* was used with the aim of increasing soluble protein at low-temperature, a protein corresponding to the expected molecular mass was detectable in the insoluble fractions and chitinase activity (ca. 4 U per g cells) was associated with the soluble form of the recombinant protein as reported for the pET24b(+) plasmid (Supplementary Fig. S3).

a

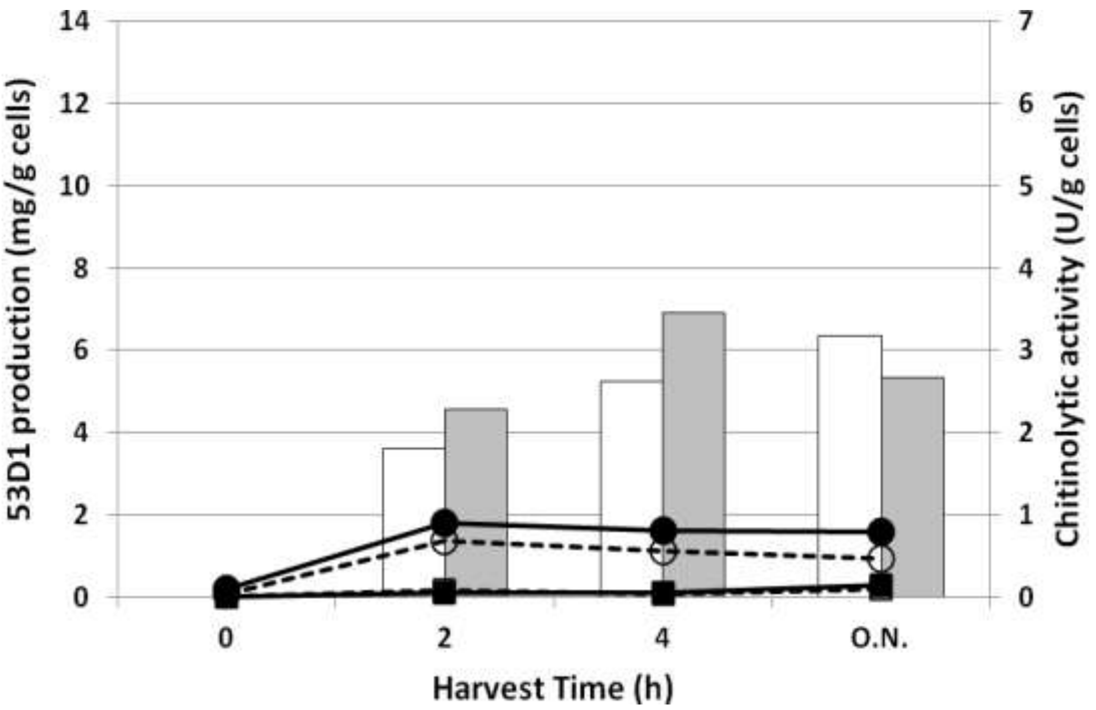

b

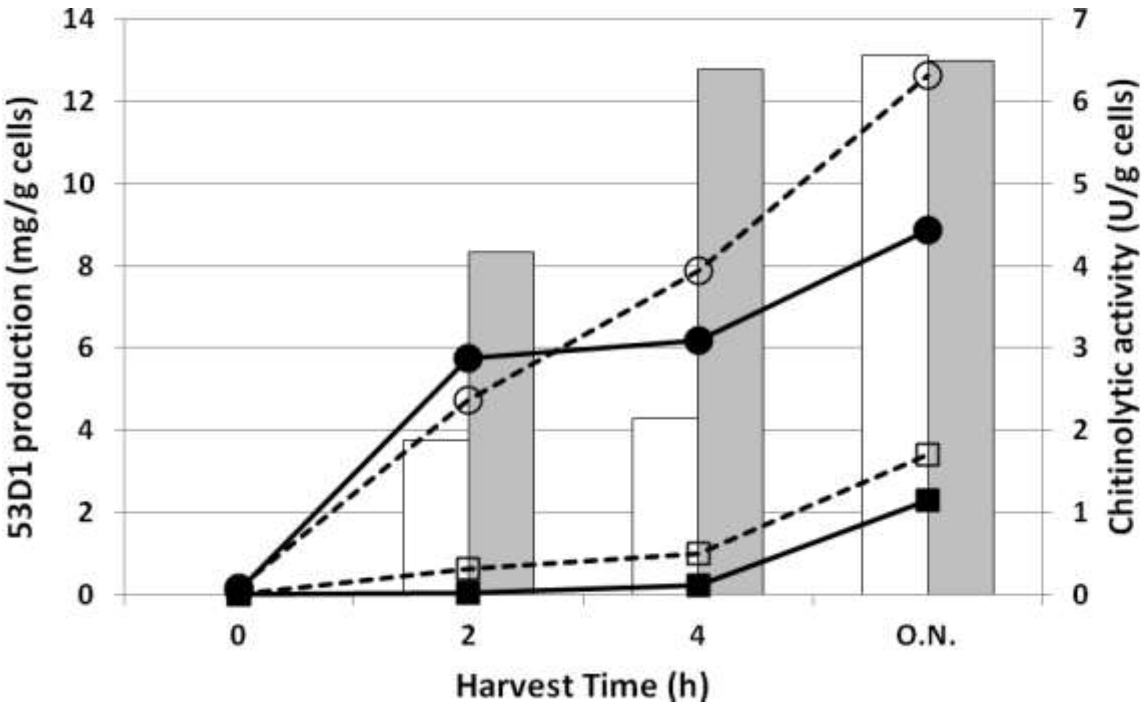

Supplementary Figure S2. 53D1 expression levels in *E. coli* BL21 Star^{TM}(DE3)/ pET24b(+)::*53D1* recombinant strain. The recombinant strain was incubated at 37°C (a) or 25°C (b) after induction with 0.5 mM IPTG and cells were harvested after 0, 2, 4 h and overnight (O.N.) from induction. 53D1 production into insoluble fractions (expressed as milligram of protein per gram of cells in wet weight) was determined by Western blot analysis using an anti-His-tag monoclonal antibody in cells

grown in LB (empty bars) or TB (grey bars). Chitinase activity was measured by fluorimetric assay on 4-MU-$(GlcNAc)_2$, either in the soluble cytoplasmic fractions of cells grown in LB (○, dashed line) or TB (●, solid line) or in the insoluble fractions (membrane and inclusion bodies) of cells grown in LB (□, dashed line) or TB (■, solid line).

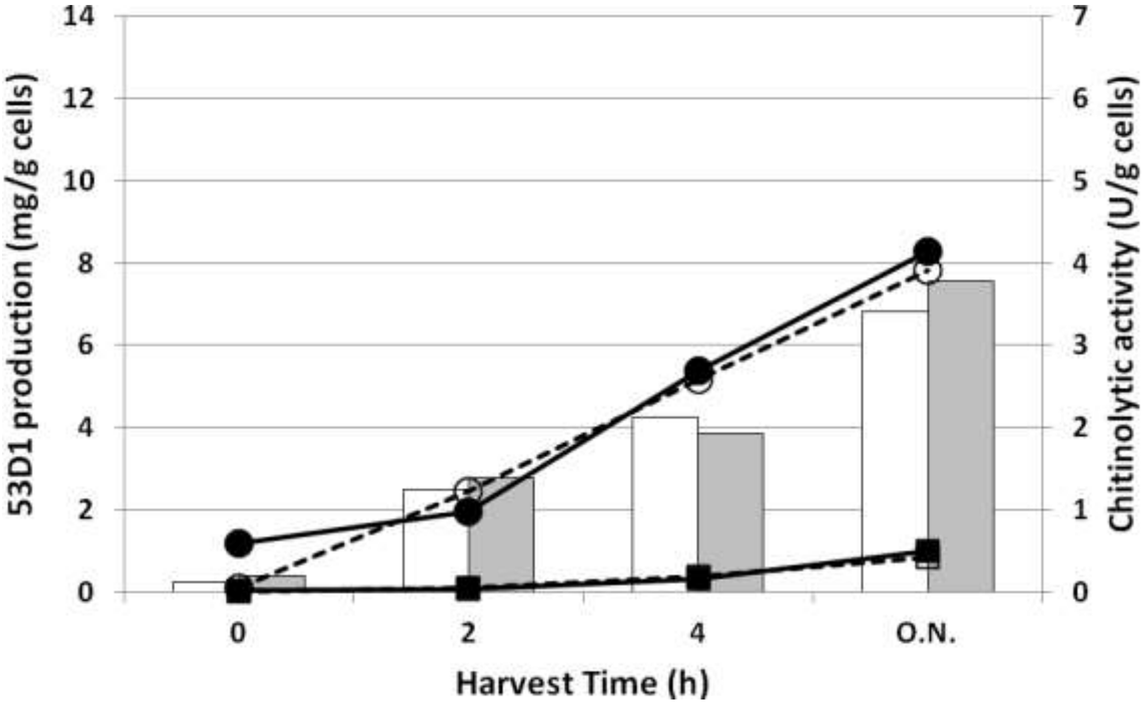

Supplementary Figure S3. 53D1 expression levels in *E. coli* BL21Star^{TM}(DE3)/pCOLDI::*53D1* recombinant strain. The recombinant strain was incubated at 15°C after induction with 0.5 mM IPTG and cells were harvested after 0, 2, 4 h and overnight (O.N.) from induction. 53D1 production into insoluble fractions (expressed as milligram of protein per gram of cells in wet weight) was determined by Western blot analysis using anti-His-tag monoclonal antibody in cells grown in LB (empty bars) or TB (grey bars). Chitinase activity was measured by fluorimetric assay on 4-MU-$(GlcNAc)_2$, either in the soluble cytoplasmic fractions of cells grown in LB (○, dashed line) or TB (●, solid line) or in the insoluble fractions (membrane and inclusion bodies) of cells grown in LB (□, dashed line) or TB (■, solid line).

References

Gornall AG, Bardawill CJ, David MM (1949) Determination of serum proteins by means of the biuret reaction. J Biol Chem 177:751-766

Job V, Marcone GL, Pilone MS, Pollegioni L (2002) Glycine oxidase from *Bacillus subtilis*. Characterization of a new flavoprotein. J Biol Chem 277:6985-6993

Hjort K, Presti I, Elväng A, Marinelli F, Sjöling S (2014) Bacterial chitinase with phytopathogen control capacity from suppressive soil revealed by functional metagenomics. Appl Microbiol Biotechnol 98:2819-2828

Supplementary Tables

Table S1: Functional annotation criteria. (a) BLASTP settings according to protein size (amino acid residues); (b) BLASTP criteria used to validate the affiliation of ORFs to specific proteins

a

BLAST settings	*Query size (amino acids residues)*			
	>85 residues	***50-85 residues***	***35-50 residues***	***<35 residues***
Weight matrix	BLOSUM* 62	BLOSUM 80	PAM*70	PAM30
Gap cost	11,1	10,1	10,1	9,1
Low complexity filter	on	off	off	off
E value	10	10	1000	1000
Word size	3	3	3 or 2	2

*BLOSUM= Bloks Substitution Matrix. Calculates sequence comparison with less than 62% (default) or 80% identity.
*PAM=Point Accepted Mutation. Calculates sequence comparison based closely related proteins.

b

Query coverage (%)	***Maximum identity (%)***	***Aligment scores (total & maximum)***	***E-value***
100%	20-100%	best hit	$<10^{-15}$
50%	48-100%	best hit	$<10^{-10}$
30%	75-100%	best hit	$<10^{-5}$

Table S2. General characteristics of genetic fragments recovered from chitin-amended soil fosmid library. (a) Sequence information; (b) Fosmid length, gaps, G+C%, predicted ORFs

a

Fosmid	*Consensus length (bp)*	*Total read count*	*Single reads*	*Reads in pairs*	*Average coverage*
14A	22632	7547953	342043	7205910	5645,37
22G3	21255	8725007	436681	8288326	6428
28C5	31983	6426283	296701	6129582	47985,18
53D1	35473	9639420	29260	9610160	13743
101F8	37907	7863506	14887	7535920	1367,57

b

Fosmid	*Length (bp)*	*Gaps*	*G+C%*	*ORFs*			
				Total	*Non-hypothetical*	*Hypothetical*	*Unknown*
14A	22632	3	52,7	18	12	6	0
22G3	21255	1	58,8	19	11	7	1
28C5	31983	0	65,2	34	20	14	0
53D1	35473	1	54,6	34	22	11	1
101F8	37907	0	59,5	40	31	9	0

Table S3a. Annotation of fosmid 14A

Predicted source criteria: ≥50% of the best hit to one genus

CDS	*Locus*	*Gene length*	*Predicted protein (size - aminoacids, aa)*	*Best match accession no.*	*Best hit to a protein from*	*Query cover (%)*	*E-value*	*Identity (%)*
1	1203-544	660	hypothetical protein (219 aa)	ZP_02433069	*Clostridium scindens* ATCC 35704	43	0,22	42
2	1422-1769	348	hypothetical protein / phage integrase (115 aa)	ZP_06661375	*Escherichia coli* B088	84	2E-47	30
3	3164-3919	756	replication initiation protein (251 aa)	NP_061424	*Escherichia coli* K-12	47	7E-05	55
4	4507-5673	1167	plasmid-partitioning protein ParA (388 aa)	NC_010804	*Burkholderia multivorans* ATCC 17616	76	0.002	44
5	5673-6644	972	plasmid-partitioning protein ParB (323 aa)	NP_061426	*Burkholderia pseudomallei* 1710b	50	0,95	36
6	6774-7559	786	efflux ABC transporter, permease protein (261 aa)	ZP_02505309	*Burkholderia pseudomallei* BCC215	94	2E-45	27
7	7559-9952	2394	beta-D-galactosidase (797 aa)	YP_002928937	*Escherichia coli* BW2952	39	7E-12	63
8	10028-10216	189	hypothetical protein (62 aa)	ZP_14290605	*Klebsiella pneumoniae* DSM 30104	93	0,18	50
9	11480-11019	462	hypothetical protein (153 aa)	ZP_02068759	*Bacteroides uniformis* ATCC 8492	36	1,3	38
10	11606-13303	1698	putative endochitinase (565 aa)	YP_004905998	*Kitasatospora setae* KM-6054	99	0.0	99
11	13580-13927	348	phage integrase family protein (115 aa)	CP001408	*Burkholderia pseudomallei* MSHR346	91	8E-26	46
12	15322-16077	756	replication initiation protein (251 aa)	AM747720	*Burkholderia cenocepacia* J2315	98	5E-11	44
13	16665-17831	1167	plasmid-partitioning protein (388 aa)	NC_016590	*Burkholderia* sp. YI23	99	5E-41	39
14	17831-18802	972	hypothetical protein (323 aa)	NP_061426	*Burkholderia pseudomallei* K96243	17	9,8	82
15	18932-19717	786	efflux ABC transporter, permease protein (261 aa)	NC_007952	*Burkholderia xenovorans* LB400	99	1E-63	36
16	19717-20103	387	beta-mannosidase (128 aa)	NC_010943	*Stenotrophomonas maltophilia* K279a	94	2E-81	68
17	20111-22108	1998	putative sugar ABC transporter ATP-binding (665 aa)	AM747720	*Burkholderia cenocepacia* J2315	97	9E-17	54
18	22184-22372	189	hypothetical protein / phage integrase (62 aa)	ZP_06661375	*Escherichia coli* B088	39	7E-12	63

*Predicted source: *Burkholderia*-like bacteria (50% of CDS)

Table S3b. Annotation of fosmid 22G3

Predicted source criteria: ≥50% of the best hit to one phylum

CDS	***Locus***	***Gene length***	***Predicted protein (size - aminoacids, aa)***	***Best match accession no.***	***Best hit to a protein from***	***Query cover (%)***	***E-value***	***Identity (%)***
1	330-43	288	hypothetical protein (95 aa)	NC_008536	Candidatus *Solibacter usitatus*	86	6,00E-05	29
2	334-2412	2079	ABC transporter (692 aa)	YP_002977039	Candidatus *Koribacter versatilis* Ellin345	43	4,00E-80	50
3	2413-2550	138	hypothetical protein, partial (45 aa)	WP_000469023	*Shigella flexneri*	100	5,00E-22	100
4	2551-4638	2088	chitinase (695 aa)	YP_002753183	*Acidobacterium capsulatum* ATCC 51196	100	0	100
5	6727-7137	411	hypothetical protein (136 aa)	NP_286554	*Shigella flexneri*	100	7,00E-95	100
6	7138-7899	762	hypothetical protein (253 aa)	NP_286553	*Escherichia coli* O157:H7 str. EDL933	100	0	100
7	7900-9141	1242	cardiolipin synthetase (413 aa)	NP_286552	*Escherichia coli*	100	0.0	100
8	9142-10098	957	conserved hypothetical protein (318 aa)	NC_012483	*Acidobacterium capsulatum* ATCC 51196	98	6,00E-105	38
9	10099-10812	714	ABC transporter inner membrane protein (237 aa)	YP_593488	Candidatus *Koribacter versatilis* Ellin345	79	5,00E-19	42
10	10873-11517	645	membrane protein (214 aa)	YP_002754337	*Acidobacterium capsulatum* ATCC 51196	98	6,00E-105	48
11	11518-13539	2022	excinuclease ABC subunit C (673 aa)	YP_005058066	*Granulicella mallensis* MP5ACTX8	59	1,3	28
12	13540-14595	1056	*N*-Acetyl-D-glucosamine ABC transport system (351 aa)	NC_012483	*Acidobacterium capsulatum* ATCC 51196	86	4,00E-101	51
13	14596-16314	1719	putative bacterial extracellular solute-binding protein (572 aa)	NZ_ACCL02000054	*Bryantella formatexigens* DSM 14469	89	2,00E-85	41
14	16315-16581	267	universal stress protein family protein (88 aa)	YP_002753605	*Acidobacterium capsulatum* ATCC 51196	97	5,00E-28	32
15	16582-18891	2310	LuxR family transcriptional regulator (769 aa)	YP_004218938	*Granulicella tundricola* MP5ACTX9	78	5,00E-38	33
16	18892-19359	468	unknown protein (155 aa)	AEU38485	*Granulicella mallensis* MP5ACTX8	26	1,00E-08	37
17	19360-20541	1182	hypothetical protein (393 aa)	YP_003320526	*Sphaerobacter thermophilus* DSM 20745	79	5,00E-19	42
18	20542-20829	288	hypothetical protein (95 aa)	AFH22375	Environmental *Halophage* eHP-23	17	9.2	82
19	20830-21255	426	non-ribosomal peptide synthetase (141 aa)	YP_007362971	*Myxococcus stipitatus* DSM 14675	36	0.060	50

*Predicted source: *Acidobacteria*-like bacteria (60% of CDS)

Table S3c. Annotation of fosmid 28C5

Predicted source criteria: ≥50% of the best hit to one phylum

CDS	*Locus*	*Gene length*	*Predicted protein (size - aminoacids, aa)*	*Best match accession no.*	*Best hit to a protein from*	*Query cover (%)*	*E-value*	*Identity (%)*
1	1-1308	1308	transcriptional regulator, LuxR family (435 aa)	ZP_06974075	*Ktedonobacter racemifer* DSM 44963	98	3E-31	41
2	1309-1596	288	hypothetical protein (95 aa)	CCF82402.1	*Nitrolancetus hollandicus* Lb	13	2.8	56
3	1597-2904	1308	transcriptional regulator, LuxR family (435 aa)	ZP_10243899	*Nitrolancetus hollandicus* Lb	99	9E-54	36
4	2905-3192	288	hypothetical protein (95 aa)	CCF82296	*Nitrolancetus hollandicus* Lb	54	7.2	28
5	3193-4500	1308	transcriptional regulator, LuxR family (435 aa)	ZP_10243899	*Nitrolancetus hollandicus* Lb	99	1E-63	36
6	4501-5079	579	conserved hypothetical protein (192 aa)	ZP_10243899	*Nitrolancetus hollandicus* Lb	94	2E-81	68
7	5080-6594	1515	transposase IS4 family protein (504 aa)	ZP_06965924	*Ktedonobacter racemifer* DSM 44963	97	9E-176	54
8	6595-6966	372	hypothetical protein (123 aa)	ZP_10246048	*Nitrolancetus hollandicus* Lb	39	7E-12	63
9	6967-7110	144	hypothetical protein (47 aa)	ZP_10246047	*Nitrolancetus hollandicus* Lb	93	0,18	50
10	7111-7716	606	transaldolase (201 aa)	ZP_09746516	*Saccharomonospora cyanea* NA-134	36	1,3	38
11	7717-8142	426	methyl-accepting chemotaxis sensory transducer (141 aa)	YP_001584157	*Burkholderia multivorans* ATCC 17616	43	0,22	42
12	8143-10098	1956	hypothetical protein (651 aa)	YP_003371273	*Pirellula staleyi* DSM 6068	84	2E-47	30
13	10273-10530	258	hypothetical protein (85 aa)	YP_006367565	*Modestobacter marinus*	47	7E-05	55
14	10531-10710	180	hypothetical protein (59 aa)	CCF82297	*Nitrolancetus hollandicus* Lb	73	1.3	44
15	10717-11241	525	hypothetical protein (174 aa)	ZP_21054152	*Xenococcus* sp. PCC 7305	50	0,95	36
16	11242-12987	1746	hypothetical protein (581 aa)	WP_001567847	*Escherichia coli*	94	2E-45	27
17	12988-13428	441	dehydrogenase/reductase family oxidoreductase (146 aa)	ZP_14469092	*Pseudomonas stutzeri* TS44	91	8E-26	46
18	13429-14631	1203	dihydroorotase (400 aa)	YP_003861823	*Maribacter* sp. HTCC2170	98	5E-113	44
19	14632-15312	681	Zn-dependent hydrolase, glyoxylase (226 aa)	ZP_18907840	*Leptolyngbya* sp. PCC 7375	99	5E-41	39

CDS	*Locus*	*Gene length*	*Predicted protein (size - aminoacids, aa)*	*Best match accession no.*	*Best hit to a protein from*	*Query cover (%)*	*E-value*	*Identity (%)*
20	15313-15492	180	hypothetical protein (59 aa)	CCF82291.1	*Nitrolancetus hollandicus* Lb	50	1.2	25
21	15493-17127	1635	molecular chaperone GroEL (544 aa)	YP_001124352	*Geobacillus thermodenitrificans* NG80-2	95	2E-80	36
22	17128-18831	1704	conserved hypothetical protein (567 aa)	CCF82397.1	*Nitrolancetus hollandicus* Lb	35	1.0	24
23	18832-20020	1188	chitinase (395 aa)	ZP_23766528	*Stenotrophomonas maltophilia* AU12-09	88	2E-89	45
24	20023-21288	1266	hypothetical protein (421 aa)	YP_003323291	*Thermobaculum terrenum* ATCC BAA-798	92	5E-45	36
25	21289-22386	1098	ABC-type sugar transport system, ATP-binding component (365 aa)	ZP_10244076	*Nitrolancetus hollandicus* Lb	99	1E-93	44
26	22387-23088	702	putative acetyltransferase (233 aa)	YP_004591376	*Enterobacter aerogenes* KCTC 2190	39	3,6	31
27	23089-24216	1128	ABC transporter (375 aa)	YP_003322712	*Thermobaculum terrenum* ATCC BAA-798	98	3E-110	48
28	24217-25341	1125	putative ABC transporter (374 aa)	ZP_10245776	*Nitrolancetus hollandicus* Lb	43	0.0002	25
29	25342-26364	1023	ABC-type sugar transport system, permease component (340 aa)	ZP_14747165	*Rhizobium* sp. CF080	97	2E-49	24
30	26365-27420	1056	*N*-Acetyl-D-glucosamine ABC transport system (351 aa)	YP_004961886	*Streptomyces bingchenggensis* BCW-1	85	8E-55	25
31	27478-29139	1662	putative extracellular solute-binding protein (553 aa)	ZP_06974409.1	*Ktedonobacter racemifer* DSM44963	42	1.0	24
32	29140-29406	267	conserved hypothetical protein (88 aa)	ZP_10244518	*Nitrolancetus hollandicus* Lb	78	0,019	35
33	29407-31716	2310	LuxR family transcriptional regulator (769 aa)	ZP_21980543	*Rhodococcus triatomae* BKS 15-14	98	7E-105	37
34	31717-31983	267	putative flavin-nucleotide-binding protein (88 aa)	ZP_10012406	*Saccharomonospora glauca* K62	53	0,0002	49

*Predicted source: *Chloroflexi*-like bacteria (53% of CDS; 38% of CDS similar to *Nitrolancetus hollandicus)*

Table S3d. Annotation of fosmid 53D1

Predicted source criteria: ≥50% of the best hit to one phylum

CDS	***Locus***	***Gene length***	***Predicted protein (size - aminoacids, aa)***	***Best match accession no.***	***Best hit to a protein from***	***Query cover (%)***	***E-value***	***Identity (%)***
1	330-43	288	transcriptional regulator, LuxR family (95 aa)	ZP_06974075	*Ktedonobacter racemifer* DSM 44963	96	1E-17	54
2	334-1641	1308	putative protein kinase/transcriptional regulator (435 aa)	ZP_10243899	*Nitrolancetus hollandicus* Lb	99	9E-54	36
3	1858-2436	579	conserved hypothetical protein (192 aa)	ZP_10245859	*Nitrolancetus hollandicus* Lb	94	3E-69	68
4	2485-3999	1515	transposase IS4 family protein (504 aa)	ZP_06965924	*Ktedonobacter racemifer* DSM 44963	97	3E-171	53
5	4733-4362	372	unknown function (123 aa)	YP_007149953	*Cylindrospermum stagnale* PCC 7417	100	2E-16	38
6	4935-4792	144	transposase IS66 (47 aa)	YP_578978	*Nitrobacter hamburgensis* X14	97	1.0	43
7	5968-5363	606	putative DNA primase (201 aa)	ZP_06965534.1	*Ktedonobacter racemifer* DSM 44963	35	1.0	24
8	6636-6211	426	non-ribosomal peptide synthetase (141 aa)	YP_007362971	*Myxococcus stipitatus* DSM 14675	30	0,89	50
9	8591-6636	1956	conserved hypothetical protein (651 aa)	WP_008478050	*Nitrolancetus hollandicus* Lb	82	9E-35	29
10	8783-8956	174	putative ABC-2 type transporter (57 aa)	ZP_10245593	*Nitrolancetus hollandicus* Lb	40	6.0	28
11	9306-9049	258	hypothetical protein (85 aa)	YP_006367565	*Modestobacter marinus*	46	6E-05	55
12	9991-9461	531	putative dehydrogenase (176 aa)	ZP_10245062	*Nitrolancetus hollandicus* Lb	10	0.2	35
13	11889-10144	1746	hypothetical protein (581 aa)	WP_008071442	*Novosphingobium nitrogenifigens*	95	5E-45	28
14	12140-12580	441	short chain dehydrogenase (146 aa)	ZP_10245022	*Nitrolancetus hollandicus* Lb	50	0.002	26
15	12580-13782	1203	hypothetical protein (400 aa)	CCF_82392	*Nitrolancetus hollandicus* Lb	75	0.045	30
16	13838-14518	681	beta-lactamase domain protein (226 aa)	ZP_10244249	*Nitrolancetus hollandicus* Lb	60	1.0	26
17	14546-14725	180	putative hydrolase (59 aa)	EFH90939	*Ktedonobacter racemifer* DSM 44963	51	0,032	29
18	14918-16552	1635	putative chaperone protein DnaK (544 aa)	NC_013525	*Thermobaculum terrenum*	63	0.005	32
19	16569-18272	1704	hypothetical protein (567 aa)	EFH90934	*Ktedonobacter racemifer* DSM 44963	56	0.002	27
20	18285-19475	1191	chitinase (396 aa)	BAF02588	uncultured bacterium	94	2E-109	48
				ZP_23766528	*Stenotrophomonas maltophilia*	87	1E-89	45
				ZP_06970122	*Ktedonobacter racemifer* DSM 44963	93	6E-81	41

CDS	*Locus*	*Gene length*	*Predicted protein (size - aminoacids, aa)*	*Best match accession no.*	*Best hit to a protein from*	*Query cover (%)*	*E-value*	*Identity (%)*
21	20756-19491	1266	hypothetical protein (421 aa)	YP_003323291	*Thermobaculum terrenum* ATCC BAA-798	92	5E-45	36
22	21866-20769	1098	ABC transporter (365 aa)	YP_003323291	*Thermobaculum terrenum* ATCC BAA-799	98	3E-97	45
23	22572-21871	702	hypothetical protein (233 aa)	CCF82422	*Nitrolancetus hollandicus* Lb	70	8.0	27
24	23705-22578	1128	sugar ABC transporter ATP-binding protein (375 aa)	YP_001637023	*Thermomicrobium roseum* DSM 5159	86	4E-101	51
25	24841-23717	1125	sugar ABC transporter ATP-binding protein (374 aa)	YP_002250459	*Dictyoglomus thermophilum* H-6-12	89	2E-85	41
26	25939-24917	1023	binding-protein-dependent transport (340)	ZP_06975261	*Ktedonobacter racemifer* DSM 44963	97	5E-28	32
27	26997-25942	1056	*N* acetyl-glucosamine transport (351 aa)	ZP_06975262	*Ktedonobacter racemifer* DSM 44964	78	5E-38	33
28	28770-27052	1719	extracellular solute-binding protein (572 aa)	YP_001635626	*Chloroflexus aurantiacus* J-10-fl	26	1E-08	37
29	28986-29252	267	putative protein- glyoxylase (88 aa)	ZP_10246475	*Nitrolancetus hollandicus* Lb	40	0.01	30
30	29932-30129	198	hypothetical protein (65 aa)	EFH90932	*Ktedonobacter racemifer* DSM 44963	52	7.2	24
31	30282-32591	2310	putative Protein kinase/transcriptional regulator (769 aa)	ZP_10243899	*Nitrolancetus hollandicus* Lb	94	4E-70	35
32	32770-33237	468	hypothetical protein (155 aa)	Q8ZY16	*Pyrobaculum aerophilum* str. IM2	65	7,3	26
33	33432-34613	1182	NHL repeat containing protein (393 aa)	EFH90640	*Ktedonobacter racemifer* DSM 44963	80	1E-02	35
34	35343-35023	321	conserved hypothetical protein (106 aa)	ZP_06588277	*Streptomyces roseosporus* NRRL	30	5	48

*Predicted source: *Chloroflexi*-like bacteria (76% of CDS; 35% of CDS similar to *Nitrolancetus hollandicus* and 29% similar to *Ktedonobacter racemifer*)

Table S3e. Annotation of fosmid 101F8

Predicted source criteria: ≥50% of the best hit to one phylum

CDS	*Locus*	*Gene length*	*Predicted protein (size - aminoacids, aa)*	*Best match accession no.*	*Best hit to a protein from*	*Query cover (%)*	*E-value*	*Identity (%)*
1	208-1221	1014	UDP-glucose 4-epimerase (337 aa)	ZP_11085591	*Aeromonas veronii* AMC35	100	0.0	99
2	1792-1292	501	hypothetical protein (166 aa)	ZP_17191914	*Aeromonas veronii* AMC36	100	4E-101	92
3	1801-2922	1122	chitinase (373 aa)	YP_005010140	*Niastella koreensis* GR20-10	100	0.0	100
4	3115-2885	231	hypothetical protein (76 aa)	ZP_11085592	*Aeromonas veronii* AMC35	84	5E-37	100
5	3283-4152	870	polysaccharide deacetylase family protein (289 aa)	YP_004625545	*Thermodesulfatator indicus* DSM 15286	100	0.0	100
6	4244-5614	1371	UDP-*N*-acetylmuramate ligase (456 aa)	ZP_11085593	*Aeromonas veronii* AMC35	100	0.0	99
7	5611-6222	612	3-octaprenyl-4-hydroxybenzoate decarboxylase UbiX (203 aa)	YP_004391328	*Aeromonas veronii* B565	100	5E-144	97
8	6846-6295	552	LitR, transcriptional regulator (183 aa)	YP_004391327	*Aeromonas veronii* B565	100	1E-123	93
9	7111-7641	531	hypoxanthine phosphoribosyltransferase (176 aa)	ZP_17187671	*Aeromonas veronii* AER39	100	3E-123	98
10	7753-8670	918	ABC-type multidrug transporter (305 aa)	ZP_12960923	*Aeromonas salmonicida* 01-B526	100	0.0	96
11	8667-9440	774	ABC transporter permease (257 aa)	YP_004391324	*Aeromonas veronii* B565	100	5E-179	99
12	9694-10113	420	methylamine utilization protein-like protein (139 aa)	YP_004391321	*Aeromonas veronii* B565	100	8E-75	96
13	10118-10645	528	hypothetical protein (175 aa)	YP_004391320	*Aeromonas veronii* B566	100	5E-124	98
14	12104-10725	1380	hypothetical protein (459 aa)	ZP_17197498	*Aeromonas veronii* AER397	98	0.0	96
15	12599-12249	351	antibiotic biosynthesis monooxygenase domain-containing protein (116 aa)	YP_004391318	*Aeromonas veronii* B565	100	7E-80	99
16	13481-12624	858	pantothenate synthetase (285 aa)	ZP_17187681	*Aeromonas veronii* AER39	100	0.0	98
17	14523-13729	795	3-methyl-2-oxobutanoate hydroxymethyltransferase (264 aa)	YP_004391316	*Aeromonas veronii* B565	100	0.0	99
18	15029-14520	510	putative pyrophosphokinase (169 aa)	YP_004391315	*Aeromonas veronii* B565	100	5E-98	96
19	16471-15041	1431	poly(A) polymerase (476 aa)	YP_858006	*Aeromonas hydrophila* ATCC 7966	95	0.0	95
20	17513-16719	795	glutamyl-queuosine tRNA(Asp) synthetase (264 aa)	ZP_11085610	*Aeromonas veronii* AMC35	100	1E-176	93

CDS	*Locus*	*Gene length*	*Predicted protein (size - aminoacids, aa)*	*Best match accession no.*	*Best hit to a protein from*	*Query cover (%)*	*E-value*	*Identity (%)*
21	18188-17739	450	RNA polymerase-binding protein DksA (149 aa)	YP_858004	*Aeromonas hydrophila* ATCC 7966	100	2E-100	95
22	19051-18326	726	sugar fermentation stimulation protein (241 aa)	ZP_11085612	*Aeromonas veronii* AMC35	100	3E-171	95
23	19575-19048	528	2'-5' RNA ligase (polyribonucleotide synthase (ATP) (175 aa)	ZP_17191893	*Aeromonas veronii* AMC34	100	2E-112	91
24	19751-21049	1299	ATP-dependent helicase HrpB (432 aa)	ZP_11085614	*Aeromonas veronii* AMC35	92	0.0	97
25	21080-21484	405	chitinase (134 aa)	NP_828094	*Streptomyces avermitilis* MA-4680	82	9E-48	77
26	21511-21786	276	Na+/H+ antiporter (91 aa)	ZP_10511762	*Bacillus mojavensis* RO-H-1	96	2E-11	40
27	21925-23136	1212	ATP-dependent helicase HrpB (403 aa)	YP_004391309	*Aeromonas veronii* B565	100	0.0	96
28	23210-25525	2316	penicillin-binding protein 1B (771 aa)	ZP_17187690	*Aeromonas veronii* AER39	100	0.0	96
29	25585-25986	402	glyoxylase I family protein (133 aa)	YP_004391307	*Aeromonas veronii* B565	100	1E-75	92
30	26372-26052	321	SMR family multidrug efflux pump (106 aa)	ZP_08521667	*Aeromonas caviae* Ae398	100	6E-45	87
31	26761-26360	402	SMR family multidrug efflux pump (133 aa)	ZP_11388620	*Aeromonas aquariorum* AAK1	100	8E-72	83
32	26919-27800	882	LysR family transcriptional regulator (293 aa)	YP_001140693	*Aeromonas veronii* B565	100	0.0	93
33	27911-29491	1581	putative sensory transduction protein (526 aa)	YP_004391303	*Aeromonas veronii* B566	100	0.0	96
34	29698-30228	531	inorganic pyrophosphatase (176 aa)	YP_001140672	*Aeromonas salmonicida* A449	100	1E-125	99
35	30604-30257	348	hypothetical protein (115 aa)	ZP_17187697	*Aeromonas veronii* AER39	100	1E-71	90
36	30744-31634	891	hypothetical protein (296 aa)	ZP_17191883	*Aeromonas veronii* AMC34	100	0.0	91
37	31855-32397	543	hypothetical protein (180 aa)	ZP_11085624	*Aeromonas veronii* AMC35	100	3E-123	97
38	32442-32741	300	hypothetical protein (99 aa)	ZP_17187700	*Aeromonas veronii* AER39	100	3E-59	89
39	32773-33357	585	hypothetical protein (194 aa)	YP_858026	*Aeromonas hydrophila* ATCC 7966	100	1E-97	84
40	33362-34051	690	antimicrobial peptide ABC transporter (229 aa)	YP_004391296	*Aeromonas veronii* B565	100	5E-158	97

*Predicted source: *Aeromonas*-like bacteria (90% of CDS)
